# Systematic *in vitro* specificity profiling reveals nicking defects in natural and engineered CRISPR–Cas9 variants

**DOI:** 10.1093/nar/gkab163

**Published:** 2021-03-21

**Authors:** Karthik Murugan, Shravanti K Suresh, Arun S Seetharam, Andrew J Severin, Dipali G Sashital

**Affiliations:** Roy J. Carver Department of Biochemistry, Biophysics & Molecular Biology, Iowa State University, Ames, IA 50011, USA; Molecular, Cellular, and Developmental Biology Interdepartmental Program, Iowa State University, Ames, IA 50011, USA; Roy J. Carver Department of Biochemistry, Biophysics & Molecular Biology, Iowa State University, Ames, IA 50011, USA; Genome Informatics Facility, Office of Biotechnology, Iowa State University, Ames, IA 50011, USA; Genome Informatics Facility, Office of Biotechnology, Iowa State University, Ames, IA 50011, USA; Roy J. Carver Department of Biochemistry, Biophysics & Molecular Biology, Iowa State University, Ames, IA 50011, USA; Molecular, Cellular, and Developmental Biology Interdepartmental Program, Iowa State University, Ames, IA 50011, USA

## Abstract

Cas9 is an RNA-guided endonuclease in the bacterial CRISPR–Cas immune system and a popular tool for genome editing. The commonly used *Streptococcus pyogenes* Cas9 (SpCas9) is relatively non-specific and prone to off-target genome editing. Other Cas9 orthologs and engineered variants of SpCas9 have been reported to be more specific. However, previous studies have focused on specificity of double-strand break (DSB) or indel formation, potentially overlooking alternative cleavage activities of these Cas9 variants. In this study, we employed *in vitro* cleavage assays of target libraries coupled with high-throughput sequencing to systematically compare cleavage activities and specificities of two natural Cas9 variants (SpCas9 and *Staphylococcus aureus* Cas9) and three engineered SpCas9 variants (SpCas9 HF1, HypaCas9 and HiFi Cas9). We observed that all Cas9s tested could cleave target sequences with up to five mismatches. However, the rate of cleavage of both on-target and off-target sequences varied based on target sequence and Cas9 variant. In addition, SaCas9 and engineered SpCas9 variants nick targets with multiple mismatches but have a defect in generating a DSB, while SpCas9 creates DSBs at these targets. Overall, these differences in cleavage rates and DSB formation may contribute to varied specificities observed in genome editing studies.

## INTRODUCTION

Cas9 is the well-studied effector protein of type II CRISPR–Cas (clustered regularly interspaced short palindromic repeats-CRISPR associated) bacterial immune systems (1,2). Cas9 is an endonuclease that uses a dual CRISPR RNA (crRNA) and trans-activating crRNA (tracrRNA) to bind dsDNA targets that are complementary to the guide region of the crRNA and adjacent to a short, conserved protospacer-adjacent motif (PAM) sequence (3,4). Two nuclease domains in Cas9, HNH and RuvC, cut the target and non-target strand respectively, generating a double-stranded break (DSB) in the dsDNA (4) with little post-cleavage trimming (5,6). The dual RNAs can be combined into a single guide-RNA (sgRNA) and the targeting region can be varied, making Cas9-sgRNA a readily programmable, two component system for use in various biotechnological applications (4,7). In particular, DSB formation followed by DNA repair can lead to changes in genomic DNA sequence, enabling genome editing following Cas9 cleavage (8,9).

Cas9 can tolerate mismatches between the crRNA and the target DNA, which is consistent with its role as a bacterial immune system effector in facilitating defense against rapidly evolving bacteriophages (10–13). Cas9 generally tolerates multiple mismatches in the PAM-distal region while PAM-proximal ‘seed’ mismatches reduce the cleavage activity (14–18). This low fidelity leads to off-target activity when used for genome editing applications, as Cas9 can create DSBs at sites with limited homology to the intended target (16,17,19). While the commonly used wildtype (WT) *Streptococcus pyogenes* Cas9 (SpCas9) can tolerate multiple mismatches in the target sequence, other naturally occurring Cas9 orthologs from *Staphylococcus aureus*, *Neisseria meningitidis* and *Campylobacter jejuni* are reported to have higher specificity in genome editing compared to SpCas9 (20–23). Many other strategies have been developed to reduce off-target activity of Cas9 (24). SpCas9 has been engineered to improve the fidelity of target cleavage activity. Some mutations were designed to reduce DNA target interactions, making the requirement for complete complementarity with the crRNA more stringent (25,26). Mutations rationally introduced in the REC domain of SpCas9 prevent conformational changes required for nuclease domain activation when a target sequence with mismatches is encountered (27,28). Bacterial screens have also been used to select high-fidelity SpCas9 variants that maintain on-target cleavage but have reduced off-target cleavage activity (29–31).

Several methods have been developed to detect and study off-target activities of Cas9 (24,32–35). However, methods that measure Cas9 off-target editing in eukaryotic cells are limited because cellular factors like nucleosomes may sequester potential cleavage sites (36,37). DNA accessibility can also vary depending on cellular processes, which may change the outcome and detection of potential Cas9 off-target editing events. These methods also rely on DSBs in the DNA generated by Cas9 or post-cleavage DNA repair and indel formation, which can vary among cell types and experiments (24,32–35). Differences in Cas9/sgRNA delivery methods and cell lines have resulted in discrepancies in the reported specificities of high-fidelity Cas9 variants (25,27,31,35).

To avoid these pitfalls, specificity studies can be performed *in vitro* to detect the native cleavage activities of Cas9 variants (6,14,15,18,38–41). Here, we used a previously established *in vitro* plasmid library cleavage assay to compare the native cleavage specificity of different Cas9 variants (42). Our method enables the detection of target sequences that may be incompletely cleaved by Cas9, leading to nicking. We tested the cleavage activity of two WT Cas9 orthologs, SpCas9 and *S. aureus* Cas9 (SaCas9), and three engineered SpCas9 variants, SpCas9 HF1 (25), hyper-accurate Cas9 (HypaCas9) (27) and Alt-R^®^ S.p. HiFi Cas9 (31) against two different target library sequences. Each of these three variants represent a version of high-fidelity Cas9 developed via different strategies discussed above. We show that SpCas9 rapidly cleaves target sequences with up to five mismatches. While the high-fidelity Cas9 variants retained cleavage activity against targets with multiple mismatches, they have reduced rates of cleavage compared to SpCas9. High-fidelity Cas9 variants also nick target sequences with multiple mismatches, resulting in incomplete DSB formation at sites that are fully cleaved by wild-type SpCas9. Overall, our study reveals a target-sequence dependent nicking defect of high-fidelity Cas9 variants that may account for increased specificity observed in genome editing studies that often rely on DSB formation to detect off-target sites.

## MATERIALS AND METHODS

### Cas9 expression vectors

Expression plasmids for SpCas9 and high-fidelity variants were purchased from Addgene. *Streptococcus pyogenes* Cas9 (SpCas9) (pMJ806) was expressed using expression plasmid pEC-K-MBP, and SpCas9-HF1 (pJSC111) and HypaCas9 (pJSC173) were expressed using expression plasmid pCT10. pMJ806, pJSC111, pJSC173 were gifts from Jennifer Doudna and/or Keith Joung (Addgene plasmid #39312; http://n2t.net/addgene:39312; RRID:Addgene_39312; Addgene plasmid # 101209; http://n2t.net/addgene:101209; RRID:Addgene_101209; Addgene plasmid #101218; http://n2t.net/addgene:101218; RRID:Addgene_101218). The gene sequence for *S. aureus Cas9* (SaCas9) was synthesized as *Escherichia coli* codon-optimized gBlocks (purchased from Integrated DNA Technologies, IDT). SaCas9 gBlocks were cloned into pSV272 with N-terminal 6X-His sequence, a maltose binding protein (MBP) and a Tobacco Etch Virus (TEV) protease cleavage site via Gibson assembly (New England Biolabs) as per the manufacturer's protocol. All sequences were verified by Sanger sequencing (Eurofins Genomics, Kentucky, USA).

### Cas9 expression and purification

All Cas9 proteins were expressed in *E. coli* BL21 (DE3) cells. Overnight cultures of the cells carrying the expression plasmid were used to inoculate 2× TY broth supplemented with corresponding antibiotics in 1:100 ratio. The antibiotics used were kanamycin at 25 μg/ml for SpCas9 (pMJ806), and at 50 μg/ml for SaCas9 (pSV272 construct) and ampicillin at 100 μg/ml for SpCas9-HF1 (pJSC111) and HypaCas9 (pJSC173). Cultures were grown at 37°C to an optical density (600 nm) of 0.5−0.6 and IPTG was added to a final concentration of 0.2 mM to induce protein expression. The incubation was continued at 18°C overnight (∼16–18 h) and harvested the next day for protein purification.

SpCas9 was purified using a previously established protocol (43). Cells were resuspended in Lysis Buffer I (20 mM Tris–HCl pH 8.0, 500 mM NaCl, 10 mM imidazole, and 10% glycerol) supplemented with PMSF. A sonicator was used to lyse the cells and the lysate was centrifuged to remove insoluble material. The clarified lysate was applied to a HisPur™ Ni-NTA Resin (ThermoFisher Scientific) column. After washing the column with Lysis Buffer I, the bound protein was eluted in Elution Buffer I (Lysis Buffer I + 250 mM imidazole final concentration). The Ni-NTA column eluent was concentrated and run on a HiLoad 16/600 Superdex 200 gel filtration column (GE Healthcare) pre-equilibrated with SEC Buffer A (20 mM Tris–HCl, pH 8.0, and 500 mM NaCl). TEV protease was added at 1:100 (w/w) ratio to the pools containing 6X His-MBP tagged Cas9 and incubated on ice, overnight at 4°C. Samples were reapplied to HisPur™ Ni-NTA Resin (ThermoFisher Scientific) to remove the His-tagged TEV, free 6X His-MBP, and any remaining tagged protein. The flow-through was collected, concentrated and further purified by using a HiLoad 16/600 S200 gel filtration column in SEC Buffer B (20 mM Tris–HCl, pH 8.0, 200 mM KCl and 1 mM EDTA). Peak pools were analyzed on SDS-PAGE gels and the pools with Cas9 were combined, concentrated, flash frozen in liquid nitrogen and stored at −80°C until further use. Cleavage activity of SpCas9 purified using this protocol was similar to commercially available SpCas9 (data not shown).

An alternative previously established purification protocol was used for all other Cas9 variants (44), with the exception of Alt-R^®^ S.p. HiFi Cas9, which was provided by Integrated DNA Technologies (IDT). Harvested cells were resuspended in Lysis Buffer II (20 mM Tris–HCl pH 8.0, 500 mM NaCl, 5 mM imidazole), supplemented with protease inhibitors (PMSF, cOmplete™ Protease Inhibitor Cocktail Tablet or Halt Protease Inhibitor Cocktail). A sonicator was used to lyse the cells and the lysate was centrifuged to remove insoluble material. The clarified lysate was applied to a HisPur™ Ni-NTA Resin (ThermoFisher Scientific) column. After washing the column with 10 column volumes of Wash Buffer (Lysis Buffer + 15 mM imidazole final concentration), the bound protein was eluted in Elution Buffer I (Lysis Buffer II + 250 mM imidazole final concentration). Fractions containing Cas9 were pooled and TEV protease was added in a 1:100 (w/w) ratio and dialyzed in Dialysis Buffer (10 mM HEPES–KOH pH 7.5, 200 mM KCl, 1 mM DTT) at 4°C overnight. The dialyzed protein was diluted 1:1 with 20 mM HEPES KOH (pH 7.5) and loaded on a HiTrap Heparin HP (GE Healthcare) column and washed with Buffer A (20 mM HEPES–KOH pH 7.5, 100 mM KCl). The protein was eluted with Buffer B (20 mM HEPES–KOH pH 7.5, 2 M KCl) by applying a gradient from 0% to 50% over a total volume of 60 ml. Eluted peak fractions were analyzed by SDS-PAGE and fractions with Cas9 were combined and concentrated. DTT was added to a final concentration of 1 mM. The protein was fractionated on a HiLoad 16/600 Superdex 200 gel filtration column (GE Healthcare), eluting with SEC buffer (20 mM HEPES–KOH pH 7.5, 500 mM KCl, 1 mM DTT). Peak pools were analyzed on SDS-PAGE gels and the pools with Cas9 were combined, concentrated, flash frozen in liquid nitrogen and stored at −80°C until further use.

Variations in Cas9 purification procedures could lead to differences in activity of the Cas9 variants. However, the level of purity was similar for all variants, and conditions were identical for all reactions (Supplementary Figure S1A) (see methods section—*in vitro* cleavage assay and analysis). All Cas9s were frozen as high concentration stocks (∼61–200 μM). Working stock concentrations of the proteins (5 or 10 μM) were made in SEC buffer (20 mM HEPES–KOH pH 7.5, 500 mM KCl, 1 mM DTT).

### Library creation

Target libraries were partially randomized to generate a pool of sequences containing mismatches (45). The following probability distribution function was used to determine the randomization/doping frequency,

(1) $$\begin{equation*}{{P }}\left( {{{n}},{{L}},{{f}}} \right){\rm{ }} = {\rm{ }}\frac{{{{L}}!}}{{{{n}}!\left( {{{L\ }} - {{\ n}}} \right)!}}\left( {{{{f}}^{{n}}}} \right)\left( {1{\rm{ }} - {{ f}}} \right)\left( {{{L}} - {{n}}} \right)\end{equation*}$$

where *P* is the pool of the population, *L* is the sequence length, *n* is the number of mutations/template and *f* is the probability of mutation/position (doping level or frequency). A randomization/doping frequency (*f*) of 15% results in a library containing a mixed pool of sequences of 20 nt (L) with a high representation of two to four mismatches (*n*). Single-stranded oligonucleotide libraries were ordered from IDT using hand mixed pools (https://www.idtdna.com/pages/products/custom-dna-rna/mixed-bases). For libraries with 15% randomization/doping frequency, if the target sequence has A at a given position, a mix of A:C:G:T would be dispensed in 85:5:5:5 ratio during oligonucleotide synthesis resulting in 85% A at this position and 15% of C, G or T (5% each).

The number of different mutation combinations (MM_c_) for a given number of mutations, *n*, and sequence length, *L*, regardless of the doping level/frequency is determined by

(2) $$\begin{equation*}M{M_{\rm c}} = {3^n} \frac{{{{L}}!}}{{{{n}}!\left( {{{L }} - {{ n}}} \right)!}}\end{equation*}$$

The total number of unique target sequences with a single mismatch is 60, with two mismatches is 1710, and with three mismatches is 30,780, etc. We used two library sequences that we previously tested for Cas12a (42), a modified protospacer 4 (PS4) sequence from *S. pyogenes* CRISPR locus (55% GC) and EMX1 gene target sequence (80% GC) (see Supplementary Table S1 for target sequence).

### Plasmid and nucleic acid preparation

All DNA oligonucleotides used in this study were synthesized by IDT or Thermo Scientific. RNAs (tracrRNA and crRNA) and single-stranded target or library oligonucleotides were ordered from IDT. Supplementary Table S1 lists the sequences of DNA and RNA oligonucleotides used in this study.

Gibson assembly was used to generate target (pTarget) and library (pLibrary) plasmids (46). The oligonucleotides for the targets or libraries were diluted to 0.2 μM in 1× NEBuffer 2. pUC19 vector was amplified using primers listed in Supplementary Table S1 via PCR to insert homology arms. The PCR reaction was subjected to DpnI digestion and PCR clean up (Promega Wizard SV Gel and PCR Clean-Up System), as per the manufacturer's protocol. 30 ng of PCR amplified pUC19, 5 μl of oligonucleotide (0.2 μM) and ddH_2_O to bring the volume to 10 μl were mixed with 10 μl 2× NEBuilder HiFi DNA Assembly Master mix (New England Biolabs) and incubated at 50°C for 1 h. NEB Stable competent cells were transformed with 2 μl of the assembled product, as per the manufacturer's protocol. Transformants were plated for plasmid preparation (for pTarget plasmids) or to assess transformation efficiencies (for pLibrary plasmids). For pTarget, starter cultures from individual colonies were used to inoculate 50 ml LB media with 100 μg/ml ampicillin. For pLibrary, all of cells in the outgrowth media from the transformation recovery were used to inoculate 50 ml LB with 100 μg/ml ampicillin. Cultures were grown overnight at 37°C for plasmid propagation and extraction using QIAGEN Plasmid Midi Kit. The following precautions were taken to ensure the plasmid remained supercoiled during plasmid extractions. Cells were cooled on ice before harvesting. All initial steps from lysis to neutralization for plasmid extractions were performed on ice with minimum mechanical stress. Plasmids were stored as aliquots that were used for up to 10 freeze-thaw cycles. Different pLibrary assembly reactions and preparations were used for the replicates of the *in vitro* cleavage assays (Supplementary Figure S1B). All pTarget sequences were verified by Sanger sequencing (Eurofins Genomics, Kentucky, USA). For controls, pUC19 was prepared by restriction enzyme digestion using BsaI-HF to linearize the plasmid and Nt.BspQI to nick the plasmid using the manufacturer's protocols (New England Biolabs).

### *In vitro* cleavage assay and analysis

The protocol was adapted from previously described methods (47). Cas9:tracrRNA:crRNA complex was formed by incubating Cas9 and tracrRNA:crRNA at a 1:1.5 ratio in reaction buffer (20 mM HEPES, pH 7.4, 100 mM KCl, 5 mM MgCl_2_, 1 mM DTT and 5% glycerol) at 37°C for 10 min. Cas9 RNP complex (final concentration 100 nM Cas9 and 150 nM tracrRNA:crRNA) was mixed with pTarget, pLibrary or empty plasmid (15 ng/μl, ∼9 nM) to initiate cleavage reactions at 37°C. Phenol–chloroform was used to quench reaction aliquots at 5, 10, 15, 30, 60, 300 and 1800 s for pTarget and at 1, 5, 30, 60 and 180 min for pLibrary. The aqueous layer was extracted and separated on a 1% agarose gel via electrophoresis and stained with SYBR Safe (Invitrogen) or RedSafe (Intron Bio) stain for dsDNA visualization. Excess tracrRNA:crRNA was used in cleavage assays to prevent any RNA-independent cleavage activity (48). All cleavage assays were performed in triplicate.

Bands were visualized and quantified with ImageJ (https://imagej.nih.gov/ij/). Intensities of the band (I) in the uncleaved (supercoiled - SC) and cleaved fractions (nicked – N and linearized – L) were measured. Fractions (FR) cleaved and uncleaved were calculated as follows:

(3) $$\begin{equation*}{\rm Fraction}{\rm{\ }}{\rm cleaved}{\rm{ }}\left( {{{\rm FR}_{\rm C}}} \right){\rm{ }} = \frac{{{I_{\rm N}}{\rm{ }} + {\rm{ }}{I_{\rm L}}}}{{{I_{{\rm SC}}} + {\rm{ }}{I_{\rm N}} + {\rm{ }}{I_{\rm L}}{\rm{ }}}}\end{equation*}$$

(4) $$\begin{eqnarray*} && {\rm Fraction}{\rm{\ }}{\rm uncleaved}{\rm{\ }}{\rm or}{\rm{\ }}{\rm supercoiled}{\rm{ }}\left( {{{\rm FR}_{{\rm SC}}}} \right){\rm{ }} \nonumber \\ && = \frac{{{I_{{\rm SC}}}}}{{{I_{{\rm SC}}} + {\rm{ }}{I_{\rm N}} + {\rm{ }}{I_{\rm L}}{\rm{ }}}} \end{eqnarray*}$$

(5) $$\begin{equation*}{\rm Fraction}{\rm{\ }}{\rm nicked}{\rm{}}\left( {{FR_{\rm N}}} \right) = \frac{{{I_{\rm N}}}}{{{I_{{\rm SC}}} + {\rm{ }}{I_{\rm N}} + {\rm{ }}{I_{\rm L}}{\rm{ }}}}\end{equation*}$$

(6) $$\begin{equation*}{\rm Fraction}{\rm{\ }}{\rm linearized}{\rm{ }}\left( {{{\rm FR}_{\rm L}}} \right){\rm{ }} = \frac{{{\rm{ }}{I_{\rm L}}}}{{{I_{{\rm SC}}} + {\rm{}}{I_{\rm N}} + {\rm{ }}{I_{\rm L}}{\rm{ }}}}\end{equation*}$$

The FR_SC_, FR_N_ and FR_L_ were determined for each of the time points ‘*t*’. FR for time point 0 (FR_0_) was determined for the negative control pLibrary (i.e. pLibrary run on a gel after preparation as represented in Supplementary Figure S1B).

The apparent rates of pTarget and pLibrary cleavage were determined by fitting FR_C_ to a one-phase association equation using GraphPad Prism v 8.4.3 (https://www.graphpad.com/scientific-software/prism/).

(7) $$\begin{equation*}{\rm FR}{\rm{ }} = {{\rm FR}_0}{\rm{ }} + \left( {{{\rm FR}_{{\rm final}}} - {\rm{ }}{{\rm FR}_0}} \right)(1 - {{\rm e}^{ - kt}})\end{equation*}$$

where *t* is time, FR is the appropriate FR_c_ that starts from FR_0_ and goes to FR_final_ (FR at the last time point), and *k* is the apparent rate constant.

### Library preparation for HTS

Agarose gel electrophoresis (as described above) was used to separate the plasmid library cleavage products into cleaved (linear and nicked) and uncleaved (supercoiled) products. The bands from the nicked and supercoiled pools from various time points were excised separately and were individually gel purified using QIAquick Gel Extraction Kit (Qiagen). Nextera Adapters (NEA) were designed to amplify across the target region in the pLibrary. Because the PCR primers amplified across the target region, Cas9-mediated linearization of the plasmid due to DSB formation at the target site did not yield any PCR product while Cas9 mediated nicked plasmid resulted in amplification of the target region via PCR. Standard Nextera unique indices/barcodes were used to multiplex the samples and were added to the first PCR products using another round of PCR (see Supplementary Table S1 for NEA primers). Samples were purified using QIAquick PCR Purification Kit (Qiagen) between the two PCR steps. The size of the PCR products was verified using Agilent 2100 Bioanalyzer. Pooled samples were subjected to NextSeq or MiSeq for paired-end reads of 75 cycles at Admera Health, LLC (New Jersey, USA) or Iowa State DNA Facility (Ames, IA). Samples were pooled and multiplexed to get an average of 100,000 reads per sample (Supplementary Figure S2D). To ensure coverage of each sample in a minimal number of NextSeq/MiSeq runs, we included two out of the three replicates performed for the pLibrary cleavage assays. Fifteen percent PhiX was spiked in to increase sequence diversity of the sample.

### HTS data analysis

Extraction of target sequences, read counts, and number of mismatches per target sequence from HTS data were analyzed using custom bash scripts (see associated GitHub repository: https://github.com/sashital-lab/Cas9_specificity). A simple workflow of the analysis is described in Supplementary Figure S3, adapted from our previous study on Cas12a (42). Target sequences were extracted along with the counts of the extracted target sequences and the number of mismatches. The files containing the extracted target sequences and counts are available on Iowa State University Library's DataShare (see Availability for more information). Target sequence information was imported into Microsoft Excel or R for plotting and summarizing, post command-line processing.

In each pool, the fraction of target sequences containing ‘n’ mismatches (MM) (F_n-MM_) was calculated as follows.

(8) $$\begin{equation*}{F_{n\hbox{-}{\rm MM}}} = {\rm{ }}\frac{{\begin{array}{@{}*{1}{c}@{}} {{\rm total}\ {\rm counts}\ {\rm of}\ {\rm sequences}\ {\rm with}\ }\\ {n\ {\rm mismatches}} \end{array}}}{{{\rm total}{\rm{\ }}{\rm counts}{\rm{\ }}{\rm of}{\rm{\ }}{\rm all}{\rm{\ }}{\rm sequences}{\rm{\ }}{\rm in}{\rm{\ }}{\rm the}{\rm{\ }}{\rm pool}}}\end{equation*}$$

F_n-MM_ was normalized to the fraction (FR) of DNA present in the supercoiled or nicked fraction at a given time point ‘*t*’ to generate an estimated abundance (EA) of a given set of sequences at a given timepoint. FR was calculated for each time point using Equations (3) through (6) as described above.

(9) $$\begin{equation*}{\rm{ }}{{\rm EA}_{n\hbox{-}{\rm MM}}} = {\rm{ }}({F_{n\hbox{-}{\rm MM}}}{\rm{\ }}{\rm of}{\rm{\ }}S{\rm{\ }}{\rm at}{\rm{\ }}t){\rm{*}}\left( {{\rm FR}{\rm{\ }}{\rm at}{\rm{\ }}t} \right)\end{equation*}$$

These values were plotted against number of mismatches (*n*) to generate mismatch distribution curves.

The relative abundance (enrichment and/or depletion) (RA) of a sequence containing ‘*n*’ mismatches at each time point ‘*t*’ compared to the negative control (i.e. pLibrary run on a gel after preparation as represented in Supplementary Figure S1B).

(10) $$\begin{equation*}{{\rm RA}_{\rm S}} = {\rm{ }}\frac{{{{\rm EA}_{n\hbox{-}{\rm MM}}}{\rm{\ }}{\rm of}{\rm{\ }}S{\rm{\ }}{\rm at}{\rm{\ }}t}}{{{{\rm EA}_{n\hbox{-}{\rm MM}{\rm{\ }}}}{\rm in}{\rm{\ }}{\rm pLibrary}}}\end{equation*}$$

Log-fold change in abundance was calculated as in Equation (11) for each time point ‘t’ and plotted as a heatmap to determine overall depletion or accumulation of targets containing a certain number of mismatches.

(11) $$\begin{equation*}{\rm Log}\hbox{-}{\rm{fold\ change\ in\ abundance }} = {\rm{ lo}}{{\rm{g}}_2}({{\rm RA}_{\rm S}})\ {\rm at}\ t\end{equation*}$$

The RA for the perfect target sequence (0 MM), RA_0MM_ was calculated using Equation (10), where *n* = 0 at the different time points. The RA for target sequences with 1 to 5 MM at each time point ‘*t*’, RA_1–5MM-*t*_ was calculated by summing EA for 1 to 5 MM, EA_1–5MM_ at each time point, and normalizing to the sum of EA of 1–5 MM in the negative control (i.e. pLibrary run on a gel after preparation as represented in Supplementary Figure S1B) as shown below:

(12) $$\begin{equation*}{{\rm RA}_{1 - 5{\rm MM} - t}} = \mathop \sum \limits_{n = 1}^{n = 5} \frac{{{{\rm EA}_{n - {\rm MM}}}{\rm{\ }}{\rm of}{\rm{\ }}S{\rm{\ }}{\rm at}{\rm{\ }}t}}{{{{\rm EA}_{n - {\rm MM}{\rm{\ }}}}{\rm in}{\rm{\ }}{\rm pLibrary}}}\end{equation*}$$

The relative cleaved fraction of counts for on-target and off-targets (RA_cleaved FR_) was determined by subtracting RA_0MM_ and RA_1–5MM_ values, respectively from 1 at each time point ‘*t*’, as shown below and plotted against time.

(13) $$\begin{equation*}{{\rm RA}_{{\rm cleaved}{\rm{\ }}{\rm FR}\hbox{-}{\rm on}\hbox{-}t}} = {\rm{ }}1 - {\rm{ }}{{\rm RA}_{0{\rm MM} - t}}\end{equation*}$$

(14) $$\begin{equation*}{{\rm RA}_{{\rm cleaved}{\rm{\ }}{\rm FR}\hbox{-}{\rm off}\hbox{-}t}} = {\rm{ }}1 - {\rm{ }}{{\rm RA}_{1 - 5{\rm MM} - t}}\end{equation*}$$

The specificity score (SS) for Cas9 cleavage was calculated by dividing the on-target by off-target RA_cleaved FR_ at each time point ‘t’.

(15) $$\begin{equation*}{\rm{SS }} = {\rm{ }}\frac{{{{\rm RA}_{{\rm cleaved}{\rm{\ }}{\rm FR} \hbox{-}{\rm on} - t}}}}{{{\rm{\ }}{{\rm RA}_{{\rm cleaved}{\rm{\ }}{\rm FR}\hbox{-}{\rm off} - t}}}}\end{equation*}$$

The specificity scores of SaCas9 and HF Cas9 variants were normalized to WT SpCas9 to determine relative specificity at each time point.

For the heatmaps, the estimated abundance (EA) of sequences containing a particular nucleotide (N = A, G, C, T) at a particular position (*P* = 1 to 20) for target sequences containing ‘*n*’ mismatches at each time point ‘*t*’ was calculated as above. Relative abundance (RA) was calculated by normalizing EA against the pool of DNA in the original library to eliminate variability in aberrant nicking that may have occurred for individual pLibraries in the negative control.

(16) $$\begin{eqnarray*} && {{\rm RA}_{{\rm S} - {\rm NP}}} \nonumber \\ && = {\rm{ }}\frac{{{{\rm EA}_{n - {\rm MM}}}{\rm{\ }}{\rm of}{\rm{\ }}{\rm S}{\rm{\ }}{\rm with}{\rm{\ }}{\rm N}{\rm{\ }}{\rm at}{\rm{\ }}{\rm P}{\rm{\ }}{\rm at}{\rm{\ }}t}}{{{\rm{\ }}{{\rm EA}_{n\hbox{-}{\rm MM}}}{\rm{\ }}{\rm of}{\rm{\ }}{\rm S}{\rm{\ }}{\rm with}{\rm{\ }}{\rm N}{\rm{\ }}{\rm at}{\rm{\ }}{\rm P}{\rm{\ }}{\rm in}{\rm{\ }}{\rm pLibrary}}} \end{eqnarray*}$$

For the supercoiled pool, we calculated the maximum change in relative abundance (RA) over time as max ΔRA_S-NP_ for each sequence containing a particular nucleotide (N = A, G, C, T) at a particular position (*P* = 1 to 20) for target sequences containing ‘n’ mismatches over all time points ‘*t*’ (0, 1, 5, 30, 60 and 180 min). Max ΔRA_S-NP_ is indicated as max Δ abundance in the figures for simplicity.

(17) $$\begin{eqnarray*} && {\rm Max}{\rm{\ }}\Delta {{\rm RA}_{{\rm S}\hbox{-}{\rm NP}}} \nonumber \\ && = {\rm{ Max}}\left[ {\frac{{({{\rm RA}_{{\rm S}\hbox{-}{\rm NP}}}{\rm{\ }}at{\rm{\ }}{t_{T - 1}}) - ({{\rm RA}_{{\rm S}\hbox{-}{\rm NP}}}{\rm{\ }}{\rm at}{\rm{\ }}{t_{\rm T}}){\rm{\ }}}}{{\left( {{t_{\rm T}}} \right) - {\rm{ }}({t_{T - 1}})}}} \right] \end{eqnarray*}$$

For the nicked pool, we calculated the average change in relative abundance (RA) over time as ΔRA_S-NP_ for each sequence containing a particular nucleotide (N = A, G, C, T) at a particular position (*P* = 1 to 20) for target sequences containing ‘*n*’ mismatches over time points ‘*t*’ after Cas9 cleavage (1, 5, 30, 60 and 180 min). ΔRA_S-NP_ is indicated as Δ abundance in the figures for simplicity.

(18) $$\begin{eqnarray*} && \Delta {{\rm RA}_{{\rm S}\hbox{-}{\rm NP}}} \nonumber \\ && = {\rm{ Average}}\left[ {\frac{{({{\rm RA}_{{\rm S}\hbox{-}{\rm NP}}}{\rm{\ }}{\rm at}{\rm{\ }}{t_T}) - ({{\rm RA}_{{\rm S}\hbox{-}{\rm NP}}}{\rm{\ }}{\rm at}{\rm{\ }}{t_{T - 1}}){\rm{\ }}}}{{\left( {{t_T}} \right) - {\rm{ }}({t_{T - 1}})}}} \right] \nonumber \\ \end{eqnarray*}$$

In the supercoiled pool, we defined the extent of cleavage of a target sequence from the supercoiled pool as abundance_min_ by determining the minimum value of RA_S-NP_ across all time points for those target sequences. For the nicked pool, we defined the extent of nicking of a target sequence as abundance_max_ by determining the maximum value of RA_S-NP_ across all time points for those target sequences. Abundance_min_ or abundance_max_ were normalized to the highest value across both pLibraries, Cas9s and mismatches (1 to 5 MM) which allows comparison between Cas9s and mismatches. Using custom scripts in R, the Δ abundance and abundance_min_ and abundance_max_ were used to plot the bubble heatmaps for the supercoiled and nicked pools, respectively. Δ max change and Δ abundance defined the gradient color and abundance_min_ and abundance_max_ defined the bubble size.

For the analysis of target sequences with two mismatches, the sequences with two mismatches were extracted. The distance between the two mismatches and the total counts for sequences separated by that distance were determined. The counts were normalized to the number of possible ways the two mismatches can occur (42), and the max Δ abundance, Δ abundance, abundance_min_ and abundance_max_ were calculated similarly to Equations (16), (17) and (18) and plotted versus distance between mismatches.

## RESULTS

### Cleavage activity of Cas9 against target library

**Figure 1.**
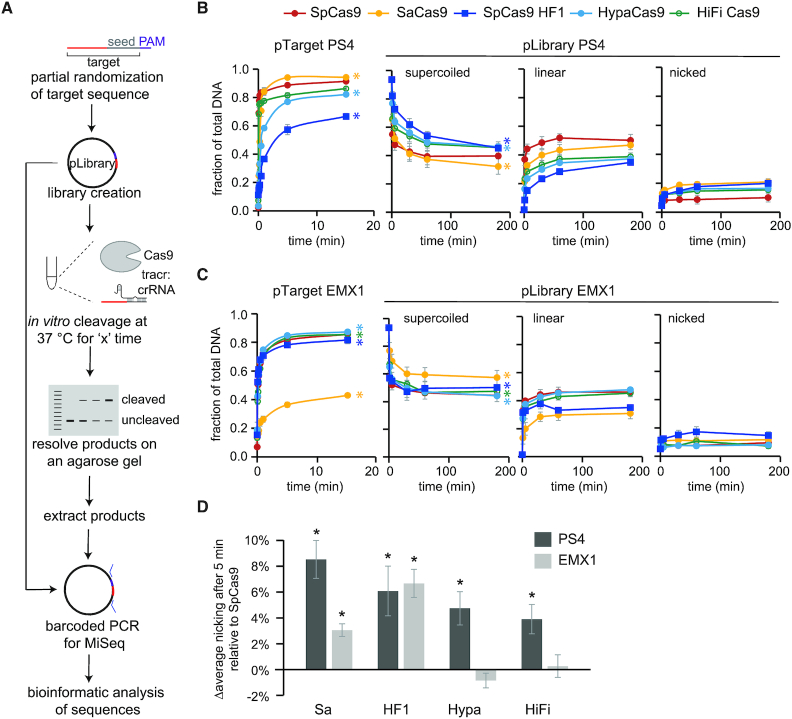
Systematic *in vitro* analysis of Cas9 mismatch tolerance. (**A**) Outline and workflow of the *in vitro* pLibrary cleavage assay. (**B**, **C**) Overall cleavage of pTarget and pLibrary (B) PS4 and (C) EMX1 by Cas9 plotted against time. Plot for pTarget shows appearance of linearized pool over time and plots for pLibrary show the decrease in supercoiled (nSC) pool and appearance of nicked (n) and linear (li) pools over time. The 0 time point is the quantification of the negative control pLibrary (i.e. pLibrary run on a gel after preparation as represented in Supplementary Figure S1B). The time points for pTarget cleavage are 5 s, 10 s, 15 s, 30 s, 1 min, 5 min, 15 min and the time points for pLibrary cleavage are 1, 5, 30, 60 and 180 min. Values plotted represent an average of three replicates for both pTarget and pLibrary. Error bars are SEM. *P < 0.05, Student's *t*-test of for rates of cleavage of pTarget and pLibrary compared to SpCas9. (**D**) Average accumulation of pLibrary nicked pool for Cas9 variants compared to SpCas9 over time. Values plotted represent an average of three replicates. Error bars are SEM. *P < 0.05, Student's *t*-test compared to SpCas9. Sa = SaCas9, HF1 = SpCas9 HF1, Hypa = HypaCas9, HiFi = Alt-R^®^ S.p. HiFi Cas9.

We sought to compare the cleavage activity and specificity of different Cas9 variants in a systematic manner. We performed a previously established *in vitro* plasmid library (pLibrary) cleavage assay with five Cas9 variants (42), WT SpCas9, WT SaCas9 and three high-fidelity variants of SpCas9–SpCas9 HF1, HypaCas9 and Alt-R^®^ S.p. HiFi Cas9 (HiFi Cas9) (Figure 1A, Supplementary Figure S1A) (25,27,31). The three high-fidelity variants of SpCas9 will be collectively referred to as HF Cas9 hereafter. For each Cas9 variant, we used two different crRNA sequences with partner tracrRNA and generated corresponding negatively supercoiled (nSC) plasmids containing the perfect target (pTarget) or target library (pLibrary) (see Materials and Methods section – Plasmid and nucleic acid preparation) (Supplementary Figure S1B). The pLibraries contained a distribution of target sequences with between zero and ten mismatches to the crRNA guide sequence, with a maximum representation of target sequences with two to four mismatches in the libraries (Supplementary Figure S1C). The two crRNA and library sequences were designed based on protospacer 4 sequence from *S. pyogenes* CRISPR locus (55% G/C) and EMX1 gene target sequence (80% G/C), referred to as pLibrary PS4 and pLibrary EMX1 respectively. We employed the native dual crRNA and tracrRNA system for our assay to avoid any differences that may stem from single guide RNA design optimization (49,50).

We used the differential migration of the nicked (n) and linear (li) cleavage products of negatively supercoiled (nSC) dsDNA plasmid on an agarose gel to analyze Cas9 cleavage activity (51) (Supplementary Figure S2A). Linear products represent fully cleaved DNA, in which both strands were cleaved by Cas9. The accumulation of linear DNA over time was used to determine rates of cleavage for pTarget. Cleavage rates of pTarget were significantly variable depending both on target sequence and Cas9 variant (Figure 1B, C, Supplementary Figure S2A, B). SpCas9 cleaved pTarget PS4 ∼3.6-fold faster than pTarget EMX1. A similar trend was observed for SaCas9, although this ortholog cleaved both pTargets ∼7-fold slower than SpCas9 (Figure 1B, C, Supplementary Figure S2A, B). Among the HF Cas9s, HiFi Cas9 had cleavage rates that were comparable to WT SpCas9. In contrast, SpCas9 HF1 and HypaCas9 cleaved pTarget PS4 ∼36- and ∼12-fold slower than SpCas9, respectively (Figure 1B, C, Supplementary Figure S2B), similar to previously reported cleavage defects for these two HF Cas9 variants (52). However, cleavage rates for SpCas9 HF1 and HypaCas9 were comparable to SpCas9 for pTarget EMX1 (Figure 1C, Supplementary Figure S2A, B), indicating that cleavage defects for HF Cas9 variants may vary based on target sequence.

For pLibrary cleavage assays, we observed a substantial amount of nicked product, resulting from incomplete cleavage of the target. We therefore determined the apparent rate of overall cleavage (nicked and linearized product) (Figure 1C, Supplementary Figure S2A, B, see methods section—*in vitro* cleavage assay and analysis). As expected, rates of pLibrary cleavage were substantially slower than for pTargets, due to the presence of mismatches in the target sequence (Figure 1B, C, Supplementary Figure S2A, B). SpCas9 rapidly cleaved >50% of both negatively supercoiled pLibraries, with the vast majority of product DNA becoming linearized (Figure 1B, C). In contrast, for SaCas9 and HF Cas9 variants, we observed greater accumulation of nicked plasmid, especially for pLibrary PS4 (Figure 1B, C). On average, all other Cas9 variants accumulated significantly more nicked product for pLibrary PS4 than SpCas9 (Figure 1D). For pLibrary EMX1, SaCas9 and SpCas9 HF1 had significantly more accumulation of nicked product than SpCas9 (Figure 1D).

We also checked whether cleavage occurred outside of the target region during pLibrary cleavage by testing the cleavage activity of Cas9 against the empty plasmid backbone without and with the different crRNAs (Supplementary Figure S2C). The empty plasmid was minimally cleaved by Cas9-tracrRNA:crRNA, except in the case of SpCas9-EMX1 crRNA where a substantial nicked product was observed at the three hour time point. However, we do not observe similar amounts of nicking of the pLibrary EMX1 by Cas9 (Supplementary Figure S2C) and further analysis indicated that pLibrary nicking is target-sequence dependent (see below).

To determine which sequences were cleaved by Cas9 variants, we extracted the plasmid DNA from the supercoiled and nicked pools, performed barcoded-PCR amplification and multiplexed, high-throughput sequencing (HTS) to get sufficient coverage of reads for each sample (Figure 1A, Supplementary Figure S2D see methods section—library preparation for HTS). Although we were unable to sequence the linearized pool using PCR amplicon sequencing, for our analysis, we assumed that target sequences absent from both the supercoiled and nicked pools were linearized. We determined the fraction of counts for the target sequences in the HTS data and normalized this fraction with the fraction of DNA present in the pool at a given time point (Figure 1B, C) to represent an estimated abundance of given target sequences within the pool (see Materials and Methods section – HTS analysis). Here, target sequences cleaved by Cas9 were depleted from the supercoiled pool while those nicked by Cas9 were enriched in the nicked pool.

**Figure 2.**
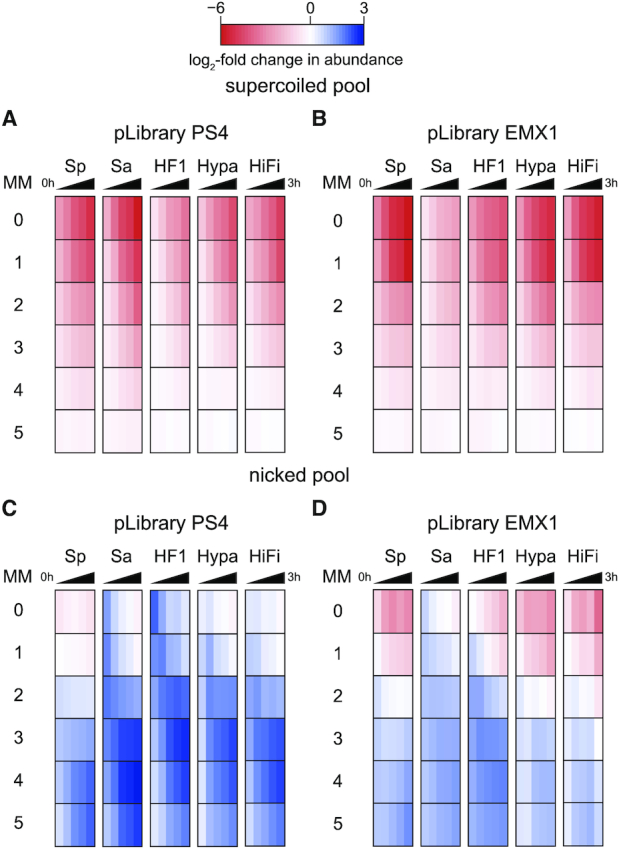
Cas9 cleavage activity against target sequences with different mismatches in pLibrary. Log_2_-fold change in abundance relative to control of target sequences containing different number of mismatches in the (**A, B**) supercoiled pool and (**C, D**) nicked pool from pLibrary (A, C) PS4 and (B, D) EMX1 when subjected to cleavage by different Cas9 variants. The time points for pLibrary cleavage are 1, 5, 30, 60 and 180 min. The color gradient represents sequences that were depleted (red), unchanged (white), or enriched (blue) relative to the control. Values plotted represent an average of two replicates. MM = mismatch, Sp = SpCas9, Sa = SaCas9, HF1 = SpCas9 HF1, Hypa = HypaCas9, HiFi = Alt-R^®^ S.p. HiFi Cas9.

We initially evaluated the cumulative effects of mismatches on the cleavage activity of each Cas9 variant by plotting the log-fold change in targets containing different numbers of mismatches with the crRNA guide sequence over time (Figure 2). We also plotted target abundance as mismatch distribution curves (Supplementary Figure S4). Together, the heatmaps and mismatch distribution curves enable overall comparison of cleavage for target sequences containing varying numbers of mismatches with the crRNA across Cas9 variants, across time points for each Cas9 variant (Figure 2, Supplementary Figure S4). As expected, the perfect target (zero mismatch) was rapidly depleted from the supercoiled pool of the pLibrary (Figure 2A, B, Supplementary Figure S4A, B). SpCas9 partially cleaved sequences with up to four mismatches in the first time point tested for both pLibraries, as observed in previous *in vitro* and *in vivo* studies on SpCas9 cleavage specificity (17,18) (Supplementary Figure S4A, B). This observation indicates that our *in vitro* pLibrary cleavage assay reproduced a similar specificity profile for SpCas9 as previous studies and can further be used to benchmark against SaCas9 and HF Cas9 variants. Like SpCas9, SaCas9 and HF Cas9 variants cleaved sequences containing up to four mismatches in both pLibraries, although the rate and extent of depletion of these sequences varied (Figure 2A, B and Supplementary Figure S4A, B). In general, variations in rates of depletion of mismatched sequences correlated with reduced rates of cleavage of the perfect target (Figure 2A, B), with SpCas9 HF1 and HypaCas9 showing slowest depletion of all targets in pLibrary PS4 and SaCas9 showing slowest depletion of all targets in pLibrary EMX1.

We also observed substantial accumulation of nicked target sequences with two to five mismatches, especially for SaCas9 and HF Cas9 variants cleaving pLibrary PS4 (Figure 2C, D, Supplementary Figure S4C, D). These results suggest that SaCas9 and HF Cas9 variants are slower to fully cleave targets containing several mismatches than WT SpCas9, resulting in formation of nicks. Notably, some target sequences with one and two mismatches were initially nicked by SaCas9 or HF Cas9 variants, but subsequently depleted from the nicked pool due to completion of DSB formation (Figure 2C, D, Supplementary Figure S4C, D). In addition, we observed differential amounts of accumulation of nicked DNA for targets containing three to five mismatches between the two pLibraries (Figure 2C, D). Overall, these data suggest that Cas9 variant and crRNA sequence can affect the rate of second-strand cleavage at mismatched targets.

### Prolonged exposure reduces specificity of high-fidelity Cas9 variants

**Figure 3.**
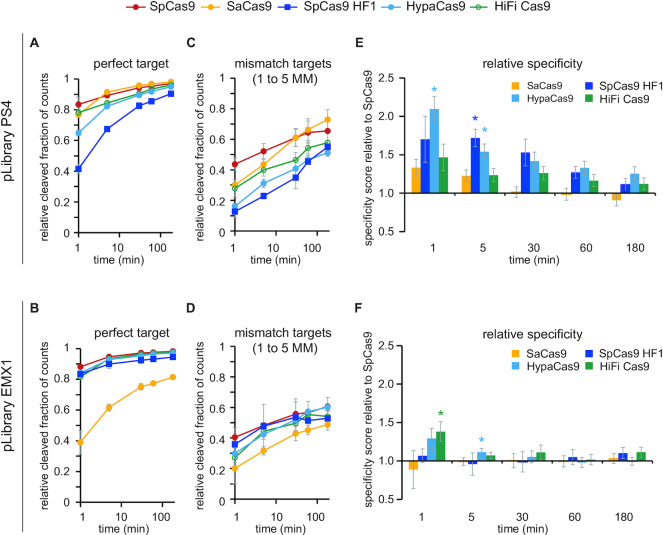
Specificity scores for Cas9 variants. (**A–D**) The fractions of the (A, B) perfect target and (C, D) target sequences with one to five mismatches (MM) that were cleaved by Cas9 variants are plotted versus time for pLibrary (A, C) PS4 and (B, D) EMX1. (E, F) Specificity scores for each Cas9 variant cleavage of pLibraries (**E**) PS4 and (**F**) EMX1 plotted relative to SpCas9 over the time course of the assay (see methods – HTS analysis). Values plotted represent an average of two replicates. Error bars are propagation of SEM. * *P* < 0.05, Student's *t*-test compared to SpCas9.

Our HTS data allows us to compare the overall cleavage efficiency and specificity of the Cas9 variants. We first determined the efficiency of cleavage of the perfect target and targets with multiple mismatches (1 to 5 MM) in the pLibrary (Figure 3A–D) (see Materials and Methods – HTS analysis). Cleavage efficiencies of the perfect target within pLibrary were similar to those observed for pTarget (Figure 1B, C, Supplementary Figure S2B). Analysis of mismatched targets indicated differences in cleavage efficiencies in comparison to the perfect target (Figure 3C, D). For example, while HiFi Cas9 cleaved the PS4 perfect target with similar efficiency to SpCas9, we observed a marked reduction in cleavage of PS4 mismatched targets for HiFi Cas9 (Figure 3C).

To analyze these differences in cleavage efficiencies, we generated a specificity score that reports the relative efficiency of cleavage of on- and off-target sequences over time for the Cas9 variants relative to SpCas9 (Figure 3E, F) (see Materials and Methods – HTS analysis). For the two WT Cas9 orthologs, we did not observe significant differences in specificity scores, suggesting that SpCas9 and SaCas9 have similar specificities for the two target sequences. All three HF Cas9 variants had some significant differences in specificity scores relative to WT SpCas9 at early time points (Figure 3E). The relative specificity scores for HF Cas9 variants were substantially larger for pLibrary PS4 than for pLibrary EMX1. Notably, we did not observe significant differences in specificity scores for any Cas9 variants at later time points (≥30 min). The lack of specificity differences between WT and HF Cas9 at longer time points indicates that prolonged exposure of HF Cas9 variants can eventually lead to off-target cleavage activity.

### Sequence determinants of Cas9 cleavage activity and nicking defects

We next wanted to characterize the effects of mismatch position and type on Cas9 cleavage (Figure 3E, F). We analyzed the sequences present in both the supercoiled and nicked pools and calculated the relative abundance of target sequences containing one to five mismatches over time (see Materials and Methods section – HTS analysis). To visualize the effects of mismatches, we used bubble heatmaps that reveal the maximal extent of cleavage (defining bubble size) and the rate of cleavage (defining gradient color) for targets containing a given mismatch type at a given position of the target.

**Figure 4.**
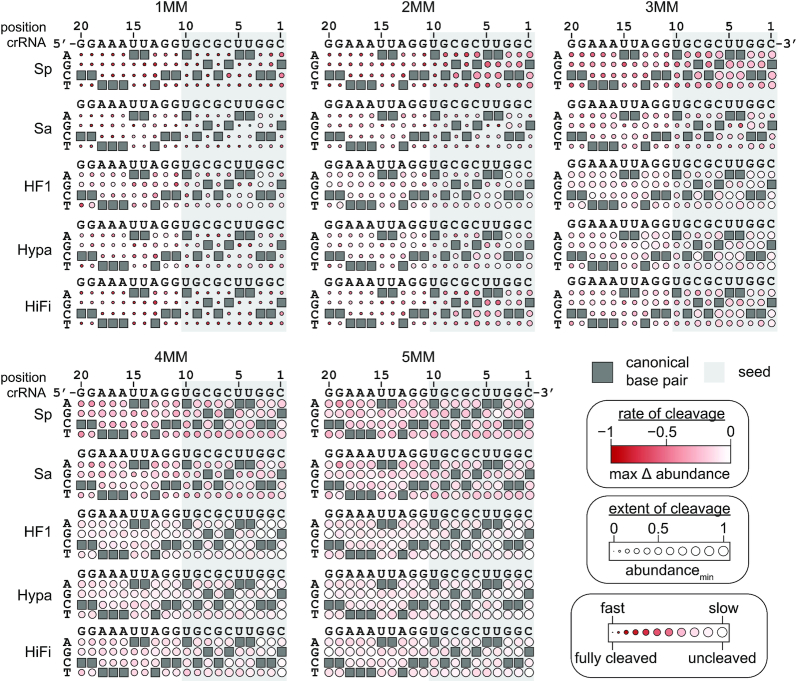
Sequence determinants of Cas9 cleavage activity for pLibrary PS4. Heatmaps showing the max Δ abundance and abundance_min_ of different mismatched sequences over time for the supercoiled pool in pLibrary PS4 upon cleavage by Cas9 variants. The position of nucleotides in the targeting region of the crRNA and the sequence are indicated on the top. The nucleotides on the left side of the heatmaps indicate the potential base pair or mismatches formed. The crRNA-complementary nucleotides are marked by grey boxes in the heatmap which result in canonical base pairs. The PAM-proximal ‘seed’ sequence is highlighted by the light grey box. The color gradient indicates sequences that were relatively depleted (red) or unchanged (white). Extent of cleavage is represented as the bubble size and varies between 0 to 1. Values plotted represent an average of two replicates. MM = mismatch.

For the supercoiled pool, target sequences that were depleted over time represent sequences that can be cleaved by Cas9. Therefore, the minimum relative abundance value in the time course (abundance_min_) represents the extent of target sequence cleavage by Cas9 (Figure 4, Supplementary Figure S5). To estimate the rate of depletion of sequences from the supercoiled pool, we calculated the maximal change in relative abundance between time points (max Δ abundance), colored as depleted (red) or unchanged (white). For SpCas9, the heatmaps reveal cleavage defects in the PAM-proximal ‘seed’ region for target sequences with two to four mismatches, similar to previously reported seed regions comprising eight to ten PAM-proximal nucleotides (6,17,18,53,54). Seed defects for target sequences with one mismatch were less pronounced. The higher tolerance for a single mismatch in the seed sequence is likely due to the relatively high concentration of Cas9 used for pLibrary cleavage (18). Notably, while seed-dependent defects were evident for other Cas9 variants (Figure 4, Supplementary Figure S5), SpCas9 HF1 and HypaCas9 also had substantial cleavage defects for targets containing mismatches located outside of the seed for pLibrary PS4 (Figure 4). Mismatches located toward the middle of PS4 (positions 11 to 13) were particularly deleterious for HypaCas9 cleaving one to three mismatch targets, while PAM-distal mismatches as far as the second to last position (position 19) from the PAM were highly deleterious for SpCas9 HF1. These results suggest that mismatches are more uniformly deleterious throughout the target for some HF Cas9 variants, although this observation was dependent on target sequence. Despite differences in the rate and extent of cleavage, mismatch specific effects were generally very similar among all Cas9 variants. These effects were more pronounced in the seed, where C–C or U–C mismatches were generally strongly deleterious (Figure 4, Supplementary Figure S5). In contrast, G–T mismatches were tolerated well within the seed for all Cas9 variants. These mismatch identity observations for Cas9 are consistent with previous *in vitro* library studies (6,40).

**Figure 5.**
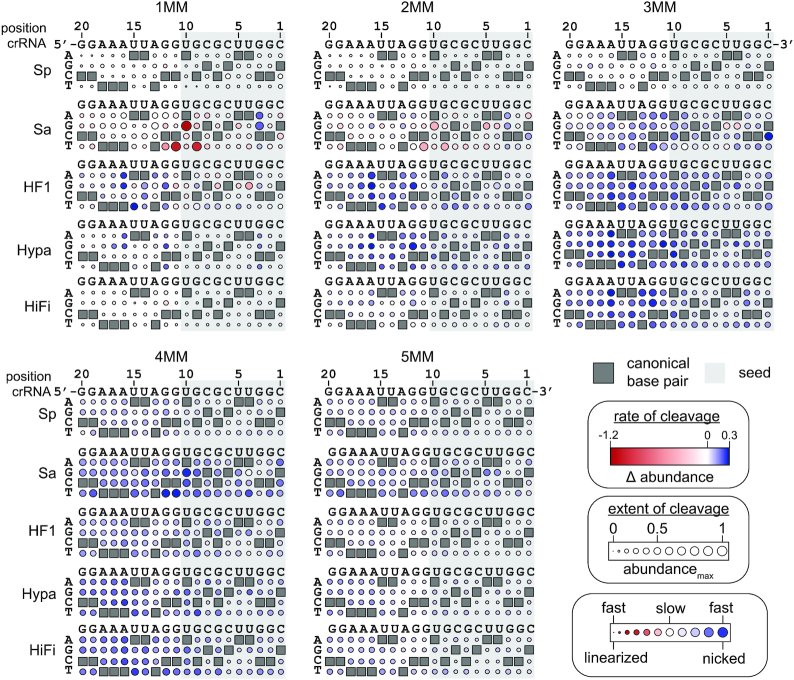
Sequence determinants of Cas9 nicking defect for pLibrary PS4. Heatmaps showing the Δ abundance and abundance_max_ of different mismatched sequences over time for the nicked pool in pLibrary PS4 upon cleavage by Cas9 variants. The position of nucleotides in the targeting region of the crRNA and the sequence are indicated on the top. The nucleotides on the left side of the heatmaps indicate the potential base pair or mismatches formed. The crRNA-complementary nucleotides are marked by grey boxes in the heatmap which result in canonical base pairs. The PAM-proximal ‘seed’ sequence is highlighted by the light grey box. The color gradient represents sequences that were depleted (red), unchanged (white), or enriched (blue) relative to the control. The extent of nicking is represented as the bubble size and varies between 0 to 1. Values plotted represent an average of two replicates. MM = mismatch.

As noted above, we observed significant accumulation of nicked plasmid for all Cas9 variants in comparison to SpCas9 for pLibrary PS4, and for SaCas9 and Cas9 HF1 for pLibrary EMX1 (Figure 1D). This accumulation was likely due to a defect in cleavage of the second strand following an initial nicking event. Our HTS data revealed that in the nicked pool, some target sequences initially have a high relative abundance that decreased over time, indicating the eventual formation of a DSB. In contrast, some target sequences were initially uncleaved but accumulated within the nicked pool over time. To visualize these effects, we plotted the maximum abundance (abundance_max_) to define the maximal extent of nicking and colored by the average change in abundance over time (Δ abundance) to define nicked targets that were depleted (red), accumulated (blue), or unchanged (white) following the first time point (Figure 5, Supplementary Figure S6). These heatmaps reveal that second strand cleavage defects are highly dependent on mismatch position, and in some cases on mismatch type. While some nicking defects for SaCas9 were caused by seed mismatches, the most notable nicking defects occurred for targets containing mismatches toward the middle of the target sequence (positions 9 to 12). For pLibrary PS4 targets, G–T or U–G mismatches within this region caused a nicking defect that was severely compounded upon addition of further mismatches. For sequences with one or two mismatches, targets containing these mismatches were initially nicked, but rapidly linearized, as visualized by large red circles (Figure 5). However, when present within three or four mismatch-containing targets, these mismatches caused the target to remain nicked for prolonged periods, as visualized by large blue circles. Similar positional defects in second-strand cleavage were observed for SaCas9 cleaving pLibary EMX1, although the effects were less dependent on mismatch type and less substantial than for PS4 (Figure 5, Supplementary Figure S6). Overall, these results suggest that mismatches toward the middle of the target can reduce second-strand cleavage by SaCas9.

For HF Cas9 variants, we observed a similar position-specific defect in second-strand cleavage for pLibrary PS4 (Figure 5, Supplementary Figure S6). These defects were not correlated with any particular mismatch type and appeared to be dependent mainly on mismatch location. Mismatches located in the PAM distal region, particularly positions 11 to 16, caused strong nicking defects for all three HF Cas9 variants for pLibrary PS4. A similar position dependence was observed for EMX1 for HF Cas9 variants, although less nicking was observed overall for this target (Figure 1D, Supplementary Figures S4D and S5). Notably, although we observed similar patterns of depletion of supercoiled DNA between SpCas9 and HiFi Cas9 (Figure 4), the mismatch position-dependent nicking defect was substantially greater for HiFiCas9 than for SpCas9, especially for PS4 targets containing three or four mismatches (Figure 5). This suggests that while HiFi Cas9 can cleave target sequences with similar numbers and types of mismatches as the wild-type protein, accumulation of mismatches in the PAM-distal region results in a defect in cleavage of the second strand for HiFi Cas9 that is not observed for the wild-type protein.

### Closely spaced mismatches compound overall and second-strand cleavage defects

For sequences containing multiple mismatches, it has previously been observed that the distance between mismatches can affect the level of cleavage defect by SpCas9 (6,14,17,18,38,39,55). We wished to determine the extent to which mismatch separation affects cleavage by all five Cas9 variants, as well as whether distance between mismatches influenced second-strand cleavage defects. We analyzed sequences containing two mismatches, which were highly represented in our target libraries (Supplementary Figure S1C). In a 20-nucleotide sequence, two mismatches can be separated by between 0 (i.e. mismatches located at adjacent positions) and 18 nucleotides (i.e. mismatches located at the beginning and end of the sequence). To determine how this distance affects the rate of cleavage for the Cas9 variants, we analyzed the supercoiled and nicked pool using bubble heatmaps as described above, but now based on the distance between the two mismatches and the location of the two mismatches.

**Figure 6.**
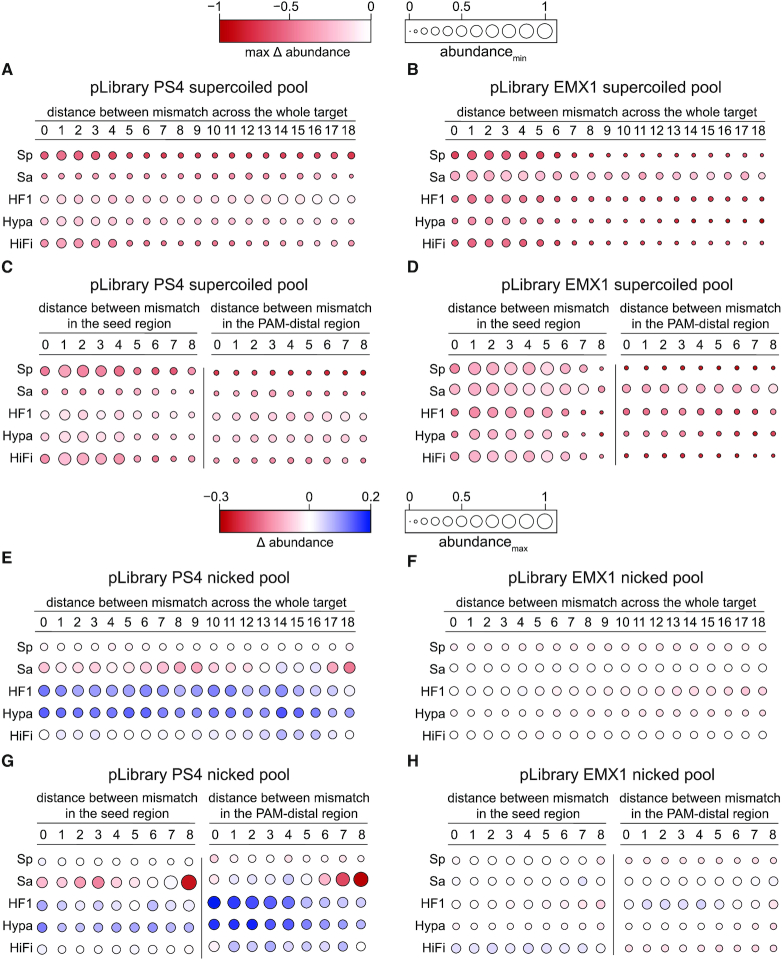
Effect of double mismatches in the target sequence on Cas9 cleavage activity. Heatmaps plotting the max Δ abundance and abundance_min_ or Δ abundance and abundance_max_ for target sequences with two mismatches in the (**A–D**) supercoiled pools and (**E–H**) nicked pools, respectively. Heatmaps show the effect of two mismatches as a function of distance between the two mismatches (A, B, E, F) across the whole target sequence or (C, D, G, H) in the seed or PAM-distal regions over time upon cleavage by Cas9 variants. The Δ max change or Δ abundance scale for supercoiled and nicked pools are indicated on the top. Abundance_min_ and abundance_max_ are represented as the bubble size and varies between 0 to 1. Values plotted represent an average of two replicates. MM = mismatch, Sp = SpCas9, Sa = SaCas9, HF1 = SpCas9 HF1, Hypa = HypaCas9, HiFi = Alt-R^®^ S.p. HiFi Cas9.

Double mismatches spaced close together (zero to four nucleotides separation) caused substantial decrease in depletion from the supercoiled pool, consistent with previous reports that closely spaced mismatches are deleterious for Cas9-dependent cleavage (17,18,38) (Figure 6A, B). One exception was SaCas9, which did not display a defect for closely spaced double mismatches for pLibrary PS4 (Figure 6A), although this defect was apparent for pLibrary EMX1 (Figure 6B). Conversely, SpCas9 HF1 displayed substantial cleavage defects for double mismatches spaced further apart (14 to 17 nucleotides separation) for PS4 (Figure 6A), a defect that was not observed for EMX1 (Figure 6B). These results underscore the variability in mismatch effects based on target sequence. To determine whether the effect of mismatch spacing is also influenced by the position within the target, we analyzed the effects of two mismatches separated by between zero and eight nucleotides within the seed or PAM-distal region (Figure 6C, D). Closely spaced mismatches (five or fewer nucleotides separation) were highly deleterious in the seed. In contrast, mismatch spacing had little impact in the PAM-distal region, where mismatches separated by any distance were similarly tolerated.

For the nicked pool, double mismatches caused similar amounts of nicking defects regardless of spacing across the whole target, as visualized by bubbles of similar sizes (Figure 6E, F). However, the rate of nicking or linearization of targets was impacted to some degree by mismatch distance. This is especially apparent for SaCas9 and HiFi Cas9 cleaving pLibrary PS4 (Figure 6E). While most double mismatches led to eventual linearization by SaCas9 and HiFi Cas9, as visualized by bubbles with shades of red or white, mismatches spaced 13 to 16 nucleotides apart were shades of blue, indicating accumulation of these targets in the nicked fraction due to a stronger nicking defect. Mismatches with this spacing necessarily places one mismatch within the PAM-distal region, consistent with the position-dependent nicking defect described above (Figure 5, Supplementary Figure S6). Further analysis of mismatch distance in the PAM-distal region revealed marked distance-dependent effects (Figure 6G, H). In general, for SaCas9 and HF Cas9 variants, mismatches spaced closer together in the PAM-distal region caused second-strand cleavage defects and accumulation in the nicked pool for pLibrary PS4 (Figure 6G). Double mismatches separated by four or fewer nucleotides in the PAM-distal region were especially deleterious for SpCas9 HF1 and HypaCas9 (Figure 6G). A similar defect for closely spaced double mismatches in the PAM-distal region was observed for SpCas9 HF1 for pLibrary EMX1, although the extent of the defect was less substantial (Figure 6H). For SaCas9 cleaving pLibrary PS4, mismatches spaced further apart (six to eight nucleotides) in either region caused a partial defect in second-strand cleavage resulting in delayed linearization, as visualized by large white or red bubbles (Figure 6G). Overall, these results indicate that multiple closely spaced mismatches within the PAM-distal region can cause reduced rates of second-strand cleavage for SaCas9 and HF Cas9 variants, albeit in a target-dependent manner.

### Validating nicking defects against mismatched targets

**Figure 7.**
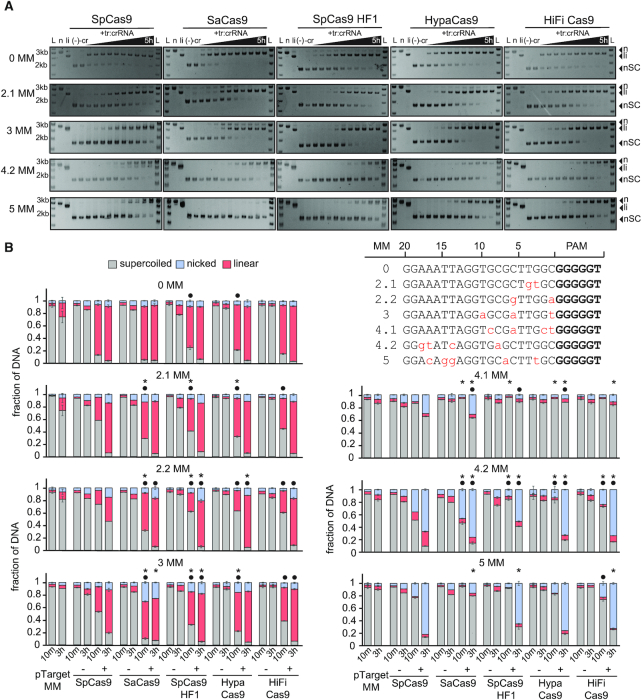
Cas9 variants have different cleavage activities against mismatched targets. (**A**) Representative agarose gels showing cleavage of a negatively supercoiled (nSC) plasmid containing the perfect target (0 MM) or mismatched (2 to 5 MM) target over a time course by Cas9 variants, resulting in linear (li) and/or nicked (n) products. Time points at which the samples were collected are 15 s, 30 s, 1 min, 2 min, 5 min, 15 min, 30 min, 1 h, 3 h and 5 h. tr:crRNA = tracrRNA:crRNA. All controls were performed under the same conditions as the longest time point for the experimental samples. Controls: (–) = pTarget or pLibrary alone incubated at 37°C for the longest time point in the assay (5 h); (-cr) = pTarget or pLibrary incubated with Cas9 only at 37°C for the longest time point in the assay (5 h); *n* = Nt.BspQI nicked pUC19; li = BsaI-HF linearized pUC19. (**B**) Quantification of supercoiled, linear and nicked pools from cleavage of perfect or fully crRNA-complementary (0 MM) and mismatched (2 to 5 MM) target plasmid by Cas9 after 10 min and 3 h. pTarget MM indicates target plasmid (0, 2 to 5 MM) alone incubated at 37°C for the time points indicated. Target sequences tested are listed with PAM (bold) and mismatches (lowercase and red) indicated. (–) indicates a cleavage reaction with the target plasmid and Cas9 only, and (+) indicates a cleavage reaction with the target plasmid, Cas9 and cognate tracrRNA:crRNA. Values plotted represent an average of three replicates. Error bars are SEM. * or • indicate *P* < 0.05 based on Student's *t*-test comparing the fraction of nicked product (*) or fraction of linear product (•) between each Cas9 variant and SpCas9.

Finally, to validate the nicking defect observed in pLibrary cleavage, we verified cleavage of individual target sequences containing two to five mismatches that were present in the nicked pool of pLibrary PS4 at the longest time point (three hours). Targets were subjected to cleavage by each Cas9 variant (Figure 7A) and the extent of nicking and linearization was quantified at 10 min and 3 h (Figure 7B). All Cas9 variants linearized targets with two or three mismatches after three hours of incubation. We observed small but significant differences in nicking and linearization between SpCas9 and other Cas9 variants for targets with two or three mismatches. The nicking defect was most notable for SaCas9 cleaving a target containing three mismatches in comparison to SpCas9, which mostly linearized this target. For targets with more than three mismatches, we observed substantially less linearization for all Cas9 variants. A target containing four mismatches within the seed (pTarget 4.1 MM) caused the strongest defect in any type of cleavage, although both SpCas9 and SaCas9 nicked 30 to 40% of the target by 3 h. Cleavage was significantly lower for all three HF Cas9 variants for this target. In contrast, all Cas9 variants cleaved target sequences with four or five mismatches in the PAM-distal region (pTarget 4.2 MM and 5 MM). As expected, based on our HTS analysis, these targets were nicked substantially but not linearized, indicating a second-strand cleavage defect. For pTarget 4.2 MM, SaCas9, HypaCas9 and HiFi Cas9 had significantly more nicked product and significantly less linearized product that SpCas9, indicating a stronger nicking defect for these variants. In contrast, SpCas9 accumulated significantly more nicked product than SaCas9, SpCas9 HF1 and HiFi Cas9 by 3 h for pTarget 5 MM, consistent with the overall lower specificity of SpCas9. Overall, these results validate stronger nicking and overall cleavage defects of SaCas9 and HF Cas9 variants in comparison to SpCas9 for the PS4 target.

## DISCUSSION

Cas9 specificity has been the subject of substantial investigation and engineering efforts, due to its importance for genome editing technologies (6,16–18,25,27,31,35,39–41). However, many previous studies investigated individual Cas9 variants separately, focusing on target binding and/or DSB formation by Cas9. Our *in vitro* library cleavage assay has enabled a comparative study of the cleavage specificity of Cas9 variants, revealing cleavage defects that have previously remained undetected (6,14,15,38,40,52). We find that engineered SpCas9 variants display higher specificity than wild-type SpCas9 in a target-dependent manner, although prolonged exposure reduces this specificity. Over time, all Cas9 variants can cleave sequences with up to five mismatches. However, while SpCas9 linearizes most target sequences with multiple mismatches as previously observed, SaCas9 and HF Cas9 variants often only nick these sequences. It is well established that Cas9 binds to sequences with limited similarity to the crRNA, although it has generally been concluded that cleavage may not occur at these sites (6,40,56–58). Our results now reveal that partial cleavage can occur at off-target sites, although second-strand cleavage defects prevent DSB formation. Most previous specificity studies tested for DSB and/or indel formation at target and off-target sites (6,25,27,31,35,39,40). Although nicked DNA may be subject to error-prone DNA repair or lead to collapse of replisomes and potential mutagenesis (59–63), nicks may also be repaired by error-free DNA repair pathways. Thus, nicking defects may obscure cleavage that does occur at off-target sites, resulting in higher genome editing specificity for SaCas9 and HF Cas9 variants.

Recent studies have compared the binding and cleavage specificities of SpCas9 and HF Cas9 variants, including Cas9 HF1 and HypaCas9 (6,52,64,65). Although target binding defects were not observed for these variants, PAM-distal mismatches decreased the rate of cleavage for both variants in comparison to SpCas9. Our results reveal that PAM-distal mismatches not only slow the rate of overall cleavage but can also slow the rate of DSB formation for SaCas9 and HF Cas9 variants, leading to nick formation. This second-strand cleavage defect may be due to R-loop collapse and premature target release following nicking of one of the strands, as has been proposed for the overall decreased kinetics of off-target cleavage by HF Cas9 variants (6,52). Additional defects may be caused by decreased movement of the HNH domain, which is required for cleavage activation of both the HNH and RuvC catalytic domains (4,27,28,66–68). Single-molecule studies of SpCas9 HF1 and HypaCas9 revealed that HNH domain movements were diminished in comparison to wild-type SpCas9, especially in the presence of PAM-distal mismatches (27). Together with our observation of nicking defects caused by PAM-distal mismatches, this suggests that cleavage by the HNH domain is impaired upon binding to targets with PAM-distal mismatches due to loss of domain rearrangements necessary to position the HNH active site for cleavage. However, sufficient HNH domain movement may occur to trigger cleavage of the non-target strand by the RuvC domain, leading to nicking of the non-target strand.

The natural role of Cas effectors is to provide defense against invading genetic elements. Specificity of these effectors has likely been tuned through evolutionary pressures exerted by rapidly evolving phages and other mobile genetic elements. Thus, it is surprising that natural orthologs of Cas effectors, including SaCas9 and various Cas12a orthologs, have been shown to have higher intrinsic genome editing specificity than SpCas9 (21,23,69–71). *In vitro* investigations have been vital for defining the native cleavage specificities of these nucleases to understand their natural role as immune effectors. We and others have observed that Cas9 and Cas12a have similar PAM-distal mismatch tolerance and similar defects for C mismatches and tolerances of T mismatch (6,42). These findings are consistent with the observation that mismatch position impacts the ability of phages to escape immunity (10,11,13), and suggest that the types of mutations that arise may be similarly consequential. We also previously observed that Cas12a, like SaCas9 and HF Cas9 variants, can cleave sequences with several mismatches, but displays a second-strand cleavage defect in the presence of multiple PAM-distal mismatches (42). The ability to nick target sequences with multiple mismatches may allow broader immunity against phages, as nicking within mutated target regions may reduce the rate of phage replication and could still enable target degradation by host nucleases (10,72). Non-specific nicking activities have also been reported for several Cas effector proteins (42,72,73), suggesting that DNA nicking is part of the vast repertoire of nucleic acid cleavage activities employed by CRISPR-Cas systems to neutralize phage infection. Future studies may determine whether single-strand breaks in the invading phage genome are sufficient for CRISPR-mediated immunity.

## DATA AVAILABILITY

HTS data and processed data files from this study have been deposited in the Iowa State University Library's DataShare and can be found at https://doi.org/10.25380/iastate.12245846. HTS data were processed with custom bash scripts which can be found at the GitHub repository https://github.com/sashital-lab/Cas9_specificity.

All other information and data are available from the authors upon request.

## Supplementary Material

gkab163_Supplemental_File

## Notes

Present address: Karthik Murugan, Integrated DNA Technologies Inc., Coralville, IA 52241, USA.
