# Supplementary material for: Systematic in vitro specificity profiling reveals nicking defects in natural and engineered CRISPR–Cas9 variants
Source: Nucleic Acids Res. 2021 Mar 21;49(7):4037–53. doi: 10.1093/nar/gkab163 (PMC8053117; doi:10.1093/nar/gkab163)
Supplement: gkab163_Supplemental_File [file gkab163_supplemental_file.pdf]

Supplementary Materials for

Systematic *in vitro* specificity profiling reveals nicking defects in natural and engineered CRISPR-Cas9 variants

Karthik Murugan [1, 2, 4], Shravanti K. Suresh [1], Arun S. Seetharam [3], Andrew J. Severin [3] and Dipali G. Sashital [1, 2 *]

Affiliations:

[1] Roy J. Carver Department of Biochemistry, Biophysics & Molecular Biology, Iowa State University, Ames, IA 50011, USA

[2] Molecular, Cellular, and Developmental Biology Interdepartmental Program, Iowa State University, Ames, IA 50011, USA

[3] Genome Informatics Facility, Office of Biotechnology, Iowa State University, Ames, IA 50011, USA

[4] Present address: Integrated DNA Technologies Inc., Coralville, IA 52241, USA

*Correspondence:

* To whom correspondence should be addressed. Tel: +1 (515)-294-5121; Fax: +1 (515)-294-7629 Email: sashital@iastate.edu

This file includes:

Figures S1 to S6

Table S1: List of Oligonucleotides

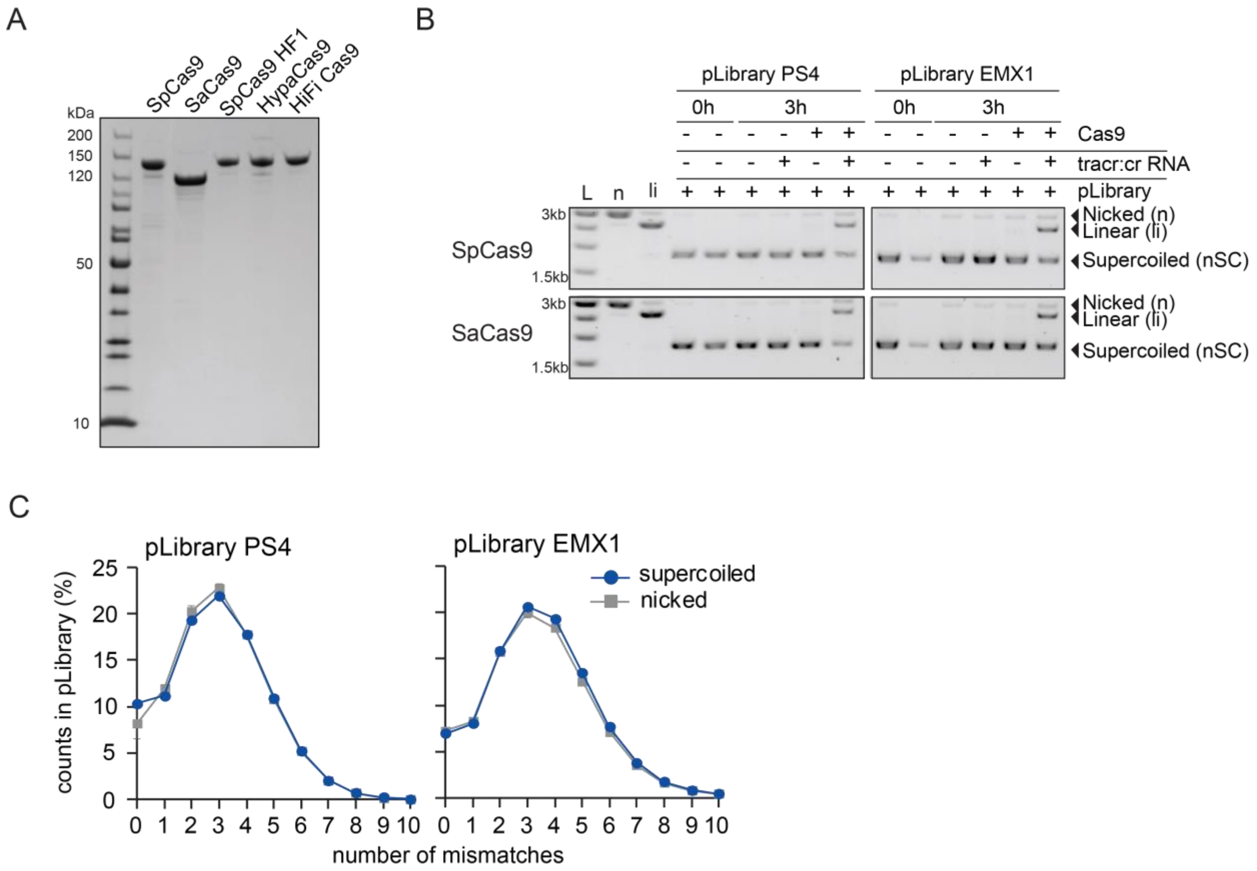

Figure S1: Components of *in vitro* pLibrary cleavage assay.

(A) Gradient SDS-PAGE of purified Cas9 variants visualized using Coomassie stain.

(B) Agarose gels showing cleavage activity of SpCas9 and SaCas9 against negatively supercoiled (nSC) plasmid library (pLibrary) only in the presence of cognate tracrRNA:crRNA, resulting in linear (li) and/or nicked (n) products after 3 hours of incubation at 37 °C. Two pLibrary replicates are labelled as 0 h and were used as the negative control pLibrary for analysis shown in panel (C).

All controls were performed under the same conditions as indicated for the longest time point for the experimental samples. n = Nt.BspQI nicked pUC19; li = BsaI-HF linearized pUC19

(C) Mismatch distribution of the supercoiled and nicked fractions from pLibrary 0 h controls (see methods section – Plasmid and nucleic acid preparation). A clear nicked plasmid band was not visible on the gel with SYBR Safe or RedSafe staining. However, a band excised from the gel in the region where the nicked fraction would run produced a similar mismatch distribution to the supercoiled fraction when subjected to HTS. This indicated the presence of trace amounts of nicked pLibrary prior to Cas9 cleavage. The mismatch distribution of this trace amount of nicked plasmid is similar to the supercoiled

fraction which means there is no sequence bias in this nicked pool and any change in the nicked pool would be a result of Cas9-mediated cleavage activity.

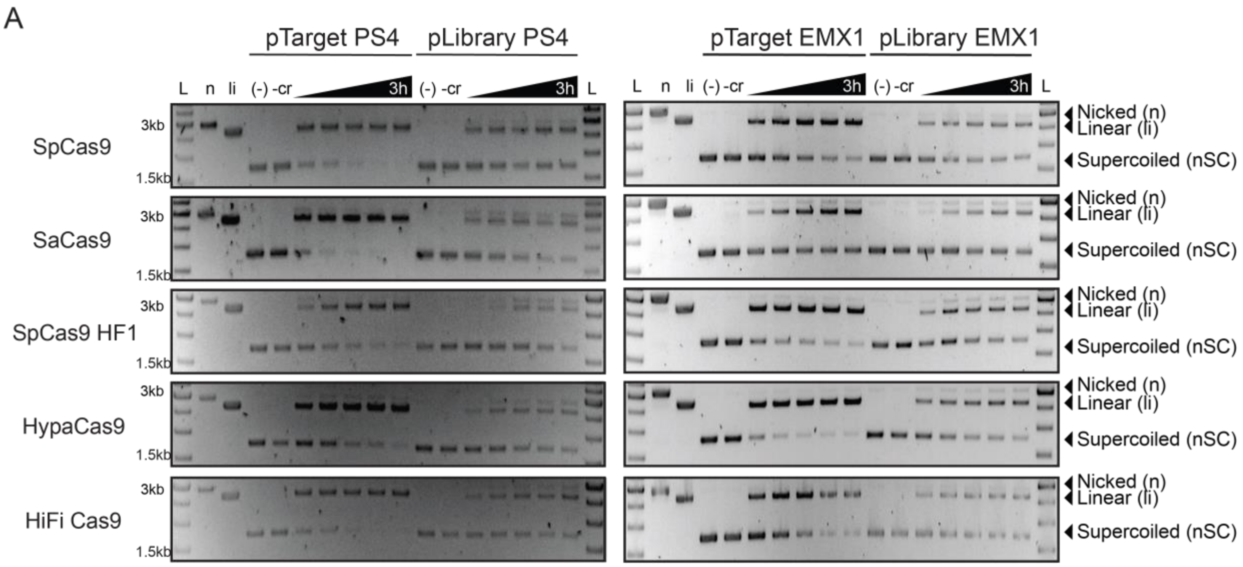

B

Cas9	apparent rates (min^{-1})			
	PS4		EMX1	
	pTarget	pLibrary	pTarget	pLibrary
Sp	18 ± 3	1.4 ± 0.2	5.0 ± 0.5	1.51 ± 0.03
Sa	2.6 ± 0.3	0.6 ± 0.3	0.7 ± 0.3	0.5 ± 0.3
HF1	0.5 ± 0.1	0.12 ± 0.05	8 ± 1	0.6 ± 0.2
Hypa	1.51 ± 0.05	0.30 ± 0.08	3.3 ± 0.5	0.97 ± 0.08
HiFi	22 ± 2	1.0 ± 0.2	2.8 ± 0.6	0.9 ± 0.1

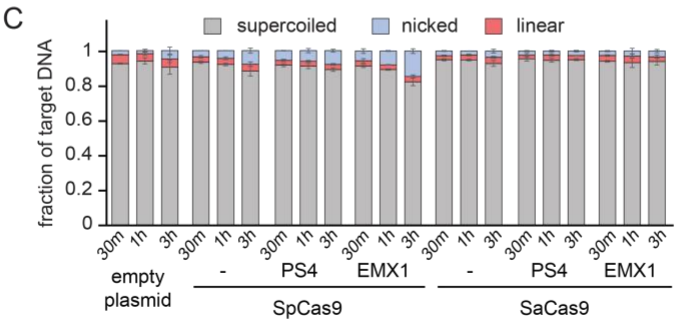

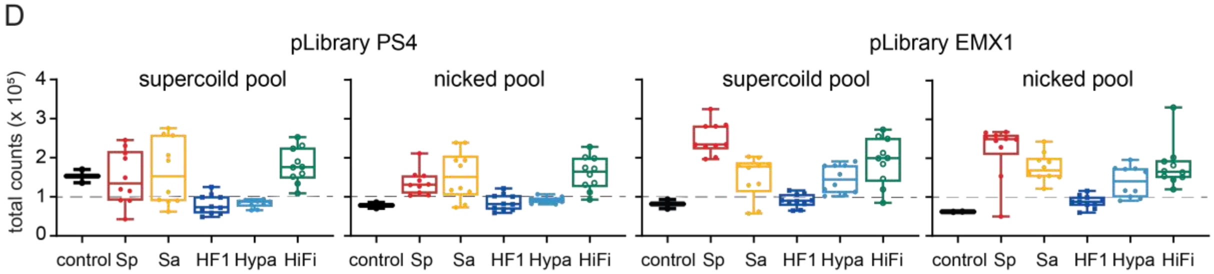

Figure S2: Cleavage activity of Cas9 on pLibrary PS4 and EMX1.

(A) Representative agarose gel showing time course cleavage of negatively supercoiled (nSC) plasmid containing a fully matched PS4 and EMX1 target (left side on each gel) and the respective plasmid library (right side on each gel) by Cas9 variants, resulting in linear (li) and/or nicked (n) products. Time points at which the samples were collected are 1 min, 5 min, 30 min, 1 h, and 3 h.

All controls were performed under the same conditions as the longest time point for the experimental samples. Controls: (-) = pTarget or pLibrary alone incubated at 37 °C for the longest time point in the assay (3 h); (-cr) = pTarget or pLibrary incubated with Cas9 only at 37 °C for the longest time point in the assay (3 h); n = Nt.BspQI nicked pUC19; li = BsaI-HF linearized pUC19

(B) Apparent rates of cleavage of pTargets and pLibraries PS4 and EMX1 by Cas9. Time points used to calculate rates were 5, 10, 15, 30, 60, 300 and 1800 s for pTarget and 1, 5, 30, 60 and 180 min for pLibrary. Values are an average of three replicates. Error bars are SEM.

(C) Quantification of supercoiled, linear and nicked fractions of empty plasmid upon incubation of Cas9 without and with different tracrRNA:crRNAs at the indicated time points at 37 °C. Values plotted represent an average of three replicates. Error bars are SEM.

(D) Box plots showing total reads from the high-throughput sequencing runs for each Cas9 variant plotted by library and product pool.

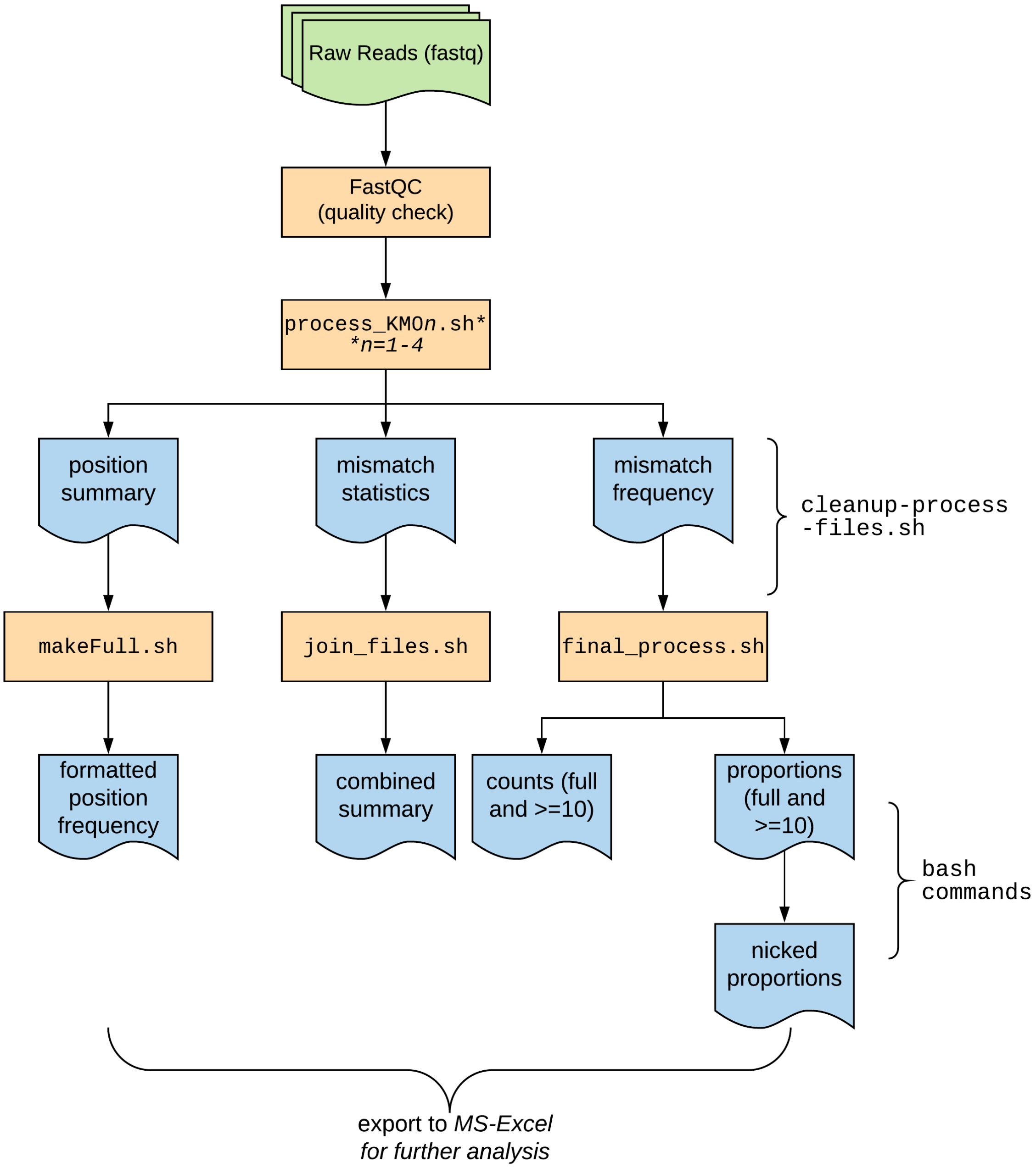

Figure S3: Workflow of the bioinformatic analysis of the HTS data.

Target sequences were extracted from the HTS data using custom scripts, previously used to study Cas12a (Murugan et al., 2020). The mismatch number and position were determined and tables reporting the counts and fractions of each mismatched target sequence were generated for further analysis (see methods – HTS data analysis)

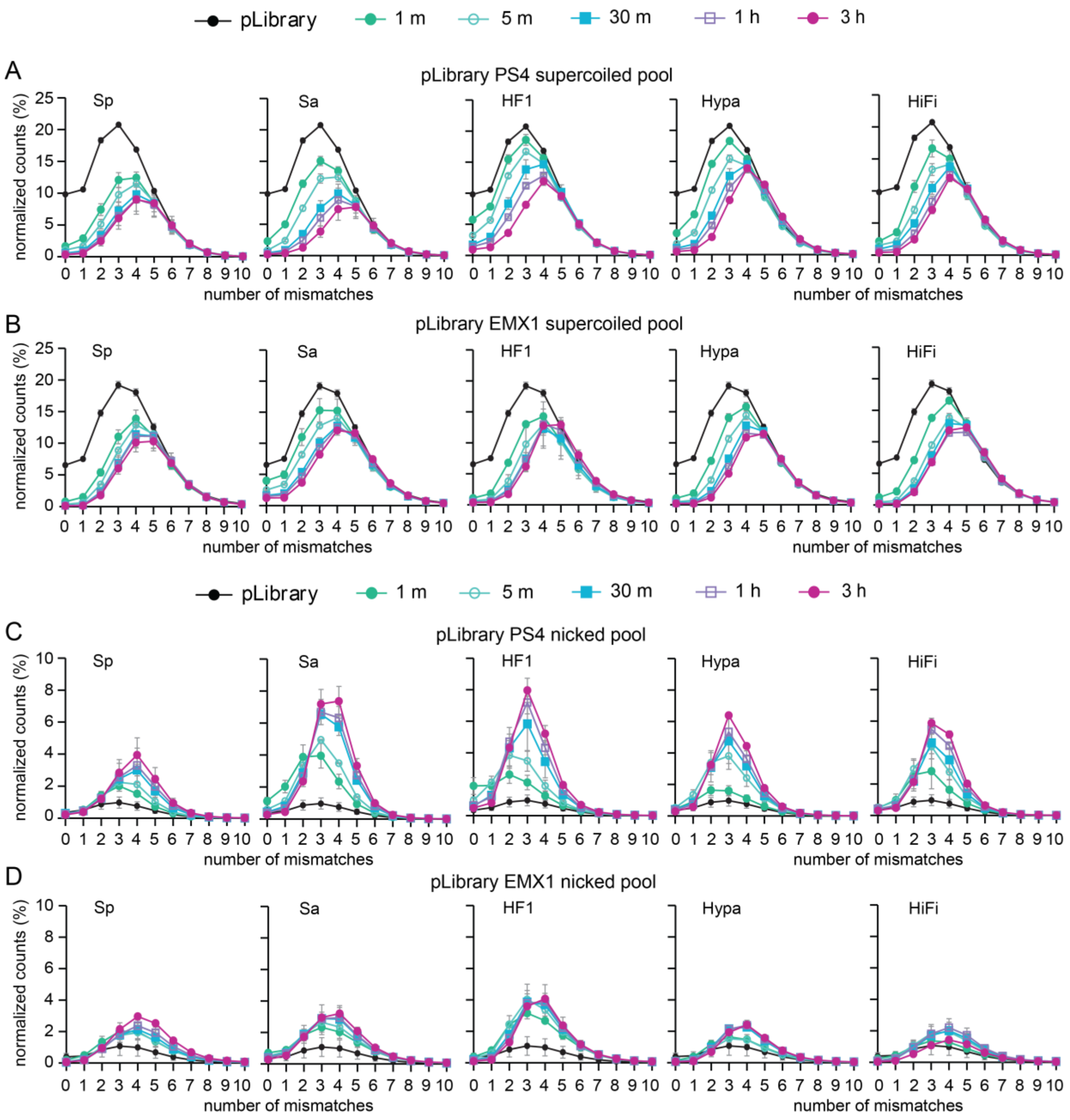

Figure S4. Mismatch distribution curves for Cas9 cleavage activity against pLibrary.

Mismatch distribution of (A, B) supercoiled pool and (C, D) nicked pool from pLibrary (A, C) PS4 and (B, D) EMX1 when subject to cleavage by different Cas9 variants. Depletion of target sequences from the supercoiled pool indicates cleavage, and enrichment in the nicked pool indicates nicking. The decrease in nicked pool over time indicates linearization of target sequences. Values plotted represent an average of two replicates. Error bars are propagation of SEM.

Sp = SpCas9, Sa = SaCas9, HF1 = SpCas9 HF1, Hypa = HypaCas9, HiFi = Alt-R ® S.p. HiFi Cas9.

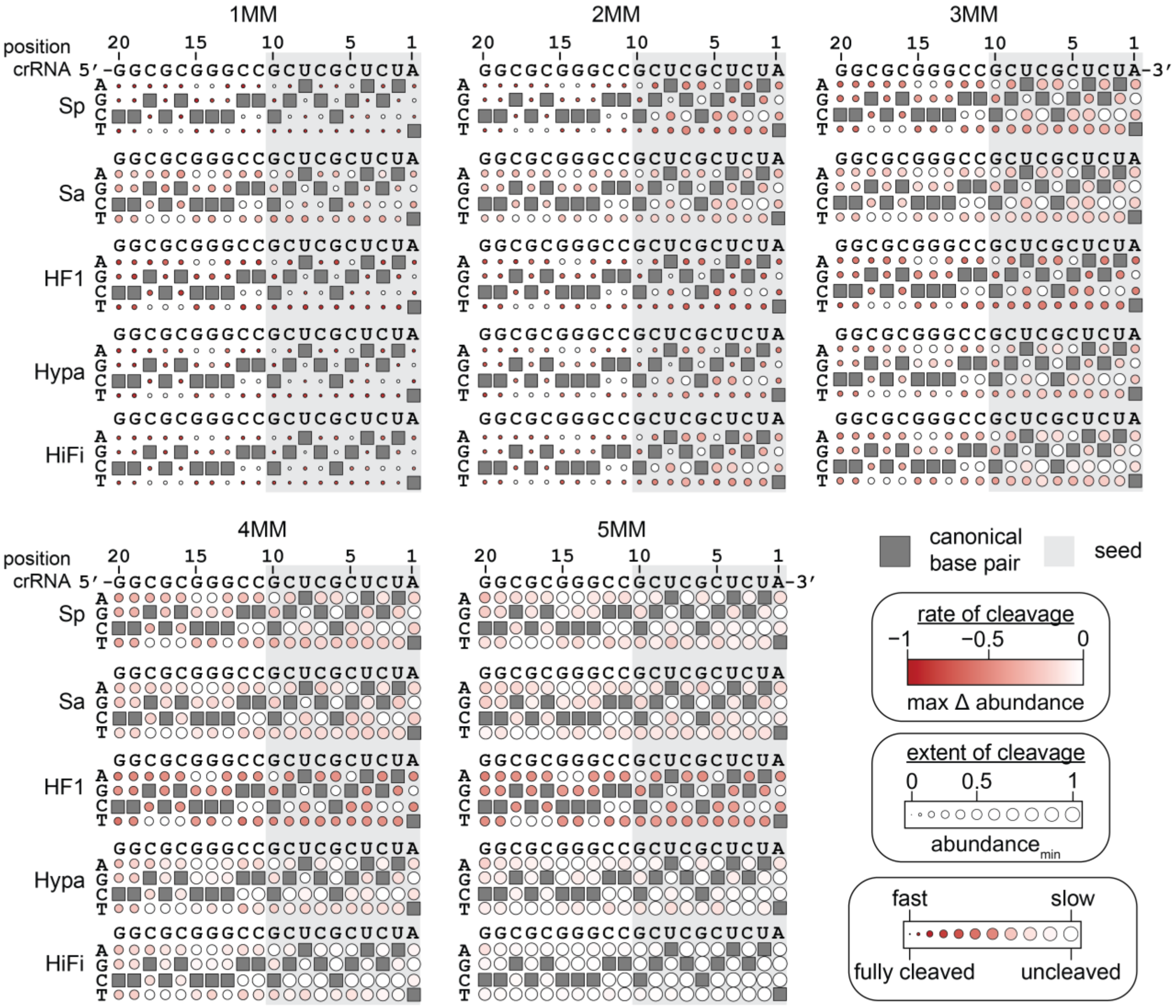

Figure S5. Sequence determinants of Cas9 cleavage activity for pLibrary EMX1.

Heatmaps showing the max Δ abundance and abundance_{min} of different mismatched sequences over time for the supercoiled pool in pLibrary EMX1 upon cleavage by Cas9 variants. The position of nucleotides in the targeting region of the crRNA and the sequence are indicated on the top. The nucleotides on the left side of the heatmaps indicate the potential base pair or mismatches formed. The crRNA-complementary nucleotides are marked by grey boxes in the heatmap which result in canonical base pairs. The PAM-proximal “seed” sequence is highlighted by the light grey box. The color gradient indicates sequences that were relatively depleted (red) or unchanged (white). Extent of cleavage is represented as the bubble size and varies between 0 to 1. Values plotted represent an average of two replicates. MM = mismatch

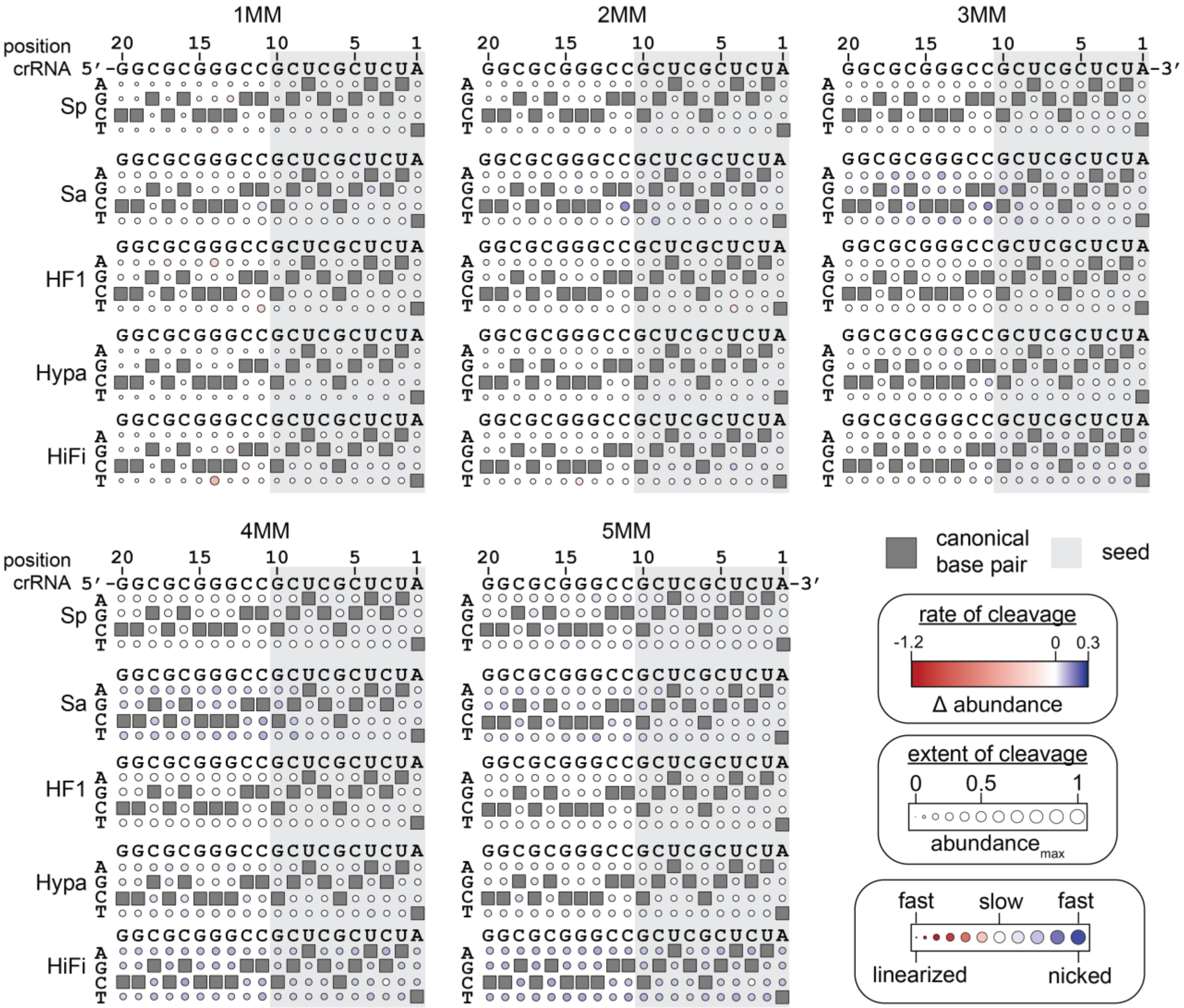

Figure S6. Sequence determinants of Cas9 nicking defect for pLibrary EMX1.

Heatmaps showing the Δ abundance and $abundance_{max}$ of different mismatched sequences over time for the nicked pool in pLibrary EMX1 upon cleavage by Cas9 variants. The position of nucleotides in the targeting region of the crRNA and the sequence are indicated on the top. The nucleotides on the left side of the heatmaps indicate the potential base pair or mismatches formed. The crRNA-complementary nucleotides are marked by grey boxes in the heatmap which result in canonical base pairs. The PAM-proximal “seed” sequence is highlighted by the light grey box. The color gradient represents sequences that were depleted (red), unchanged (white), or enriched (blue) relative to the control. The extent of nicking is represented as the bubble size and varies between 0 to 1. Values plotted represent an average of two replicates. MM = mismatch.

References:

Murugan, K., Seetharam, A.S., Severin, A.J., Sashital, D.G., 2020. CRISPR-Cas12a has widespread off-target and dsDNA-nicking effects. J. Biol. Chem. jbc.RA120.012933. https://doi.org/10.1074/jbc.RA120.012933

Table S1: List of Oligonucleotides

Key:
Bold = target sequence
underlined = PAM
lowercase = mismatch (MM)
RC = reverse complement

Sequence (5' to 3')	Notes
RNA	
GGAACCAUUCAAAACAGCAUAGCAAGUUAAAAUAAGGCUAGUCCGUUAUCAACUUGAAAAAGUGGCACCGAGUCGGUGCUUUUUUU	SpCas9 tracrRNA
AUUGUACUUAUACCUAAAAUUACAGAAUCUACUAAAACAAGGCAAAAUGCCGUGUUUAUCUCGUCAACUUGUUGGCGAGAUUUUU	SaCas9 tracrRNA
GGAAAUUAGGUGCGCUUGGCGUUUUAGAGCUAUGCUGUUUUG	SpCas9 crRNA for modified protospacer 4 (ps4) from Sp CRISPR
GGCGCGGGCCGCUCGCUCUAGUUUUAGAGCUAUGCUGUUUUG	SpCas9 crRNA for EMX1 gene target
GGAAAUUAGGUGCGCUUGGCGUUUUAGUACUCUGUAAUUUUAGGUAUGAGGUAGAC	SaCas9 crRNA for modified protospacer 4 (ps4) from Sp CRISPR
GGCGCGGGCCGCUCGCUCUAGUUUUAGUACUCUGUAAUUUUAGGUAUGAGGUAGAC	SaCas9 crRNA for EMX1 gene target
Target DNA oligonucleotides	
GCATTGCTGTACGAATCGTACAGGGTGCTTCAGGATGGAAATTAGGTGCGCTTGGCGGGGGTTGGTCAAGCTCGGACATCGTGATTGATAATGCGATGC	Cas9 modified ps4 from Sp CRISPR - 99b target - ssoligo used for Gibson assembly with pUC19 – KMlib002 – pLibrary PS4
GCATTGCTGTACGAATCGTACAGGGTGCTTCAGGTTTAGGCGCGGGCCGCTCGCTCTAGGGGGTGTCAAGCTCGGACATCGTGATTGATAATGCGATGC	EMX1 gene target - high GC % - 99b target - ssoligo used for Gibson assembly with pUC19 - KMlib003 – pLibrary EMX1
GCATTGCTGTACGAATCGTACAGGGTGCTTCAGGATGGAAATTAGGTGCGCTgtGCGGGGGTTGGTCAAGCTCGGACATCGTGATTGATAATGCGATGC	Cas9 mismatched target ssoligo - off target for mod protospacer 4, pLibrary PS4 - 2 mismatches 1 (2.1 MM)
GCATTGCTGTACGAATCGTACAGGGTGCTTCAGGATGGAAATTAGGTGCGgTTGGaGGGGGTTGGTCAAGCTCGGACATCGTGATTGATAATGCGATGC	Cas9 mismatched target ssoligo - off target for mod protospacer 4, pLibrary PS4 - 2 mismatches 2 (2.2 MM)
GCATTGCTGTACGAATCGTACAGGGTGCTTCAGGATGGAAATTAGGaGCGaTTGGtGGGGGTTGGTCAAGCTCGGACATCGTGATTGATAATGCGATGC	Cas9 mismatched target ssoligo - off target for mod protospacer 4, pLibrary PS4 - 3 mismatches 1 (3.1 MM)
GCATTGCTGTACGAATCGTACAGGGTGCTTCAGGATGGAAATTAGGTcCGaTTGctGGGGGTTGGTCAAGCTCGGACATCGTGATTGATAATGCGATGC	Cas9 mismatched target ssoligo - off target for mod protospacer 4, pLibrary PS4 - 4 mismatches 1 (4.1 MM)
GCATTGCTGTACGAATCGTACAGGGTGCTTCAGGATGGgtATcAGGTGaGCTTGGCGGGGGTTGGTCAAGCTCGGACATCGTGATTGATAATGCGATGC	Cas9 mismatched target ssoligo - off target for mod protospacer 4, pLibrary PS4 - 4 mismatches 2 (4.2

	MM)
GCATTGCTGTACGAATCGTACAGGGTGCTTCAGGATGGAcAggAGGTGCaCTTtGCGGGGGTTGGTCAAGCTCGGACATCGTGATTGATAATGCGATGC	Cas9 mismatched target ssoligo - off target for mod protospacer 4, pLibrary PS4 - 5 mismatches
Primers	
GTCAAGCTCGGACATCGTGATTGATAATGCGATGCACTGGCCGTCGTTTTACAACGTC	pUC19 plasmid library assembly - (between) M13 - vector amplification - common primer 1
CCTGAAGCACCCTGTACGATTCGTACAGCAATGCGTCATAGCTGTTTCCTGTGTGAAATTG	pUC19 plasmid library assembly - (between) M13 - vector amplification - common primer 2
TCGTCGGCAGCGTCAGATGTGTATAAGAGACAGGCATCGCATTATCAATCACGATGTC	for Nextera tagmentation - transposase adapter - forward
GTCTCGTGGGCTCGGAGATGTGTATAAGAGACAGGCATTGCTGTACGAATCGTACAGG	for Nextera tagmentation - transposase adapter - reverse
GCATTGCTGTACGAATCGTACAGGGTGCTTCAGGATGTTTACGGTTCGCGTGGtTTAtAGGTGCGTCAAGCTCGGACATCGTGATTGATAATGCGATGC	Cas12a mismatched target ssoligo - TTTA PAM - off target for mod protospacer 4, pLibrary PS4 - 2 mismatches 1 (2.2 MM)
